# Biopersistence of silver nanoparticles in tissues from Sprague–Dawley rats

**DOI:** 10.1186/1743-8977-10-36

**Published:** 2013-08-01

**Authors:** Ji Hyun Lee, Yong Soon Kim, Kyung Seuk Song, Hyun Ryol Ryu, Jae Hyuck Sung, Jung Duck Park, Hyun Min Park, Nam Woong Song, Beom Soo Shin, Daniel Marshak, Kangho Ahn, Ji Eun Lee, Il Je Yu

**Affiliations:** 1 Institute of Nanoproduct Safety Research, Hoseo University, 165 Sechul-ri, Baebang-myun, Asan 336-795 South Korea; 2 Korea Ginseng Corporation, Daejeon, South Korea; 3 Korea Conformity Laboratories, Incheon, South Korea; 4 College of Medicine, Chung-Ang University, Seoul, South Korea; 5 Korea Research Institute of Standards and Science, Daejeon, South Korea; 6 College of Pharmacy, Catholic University of Daegu, Daegu, South Korea; 7 Perkin Elmer, Waltham, MA, USA; 8 Hanyang University, Ansan, South Korea

**Keywords:** Silver nanoparticles, Biopersistence, Clearance, Size difference, Tissue distribution

## Abstract

Silver nanoparticles are known to be distributed in many tissues after oral or inhalation exposure. Thus, understanding the tissue clearance of such distributed nanoparticles is very important to understand the behavior of silver nanoparticles *in vivo*. For risk assessment purposes, easy clearance indicates a lower overall cumulative toxicity. Accordingly, to investigate the clearance of tissue silver concentrations following oral silver nanoparticle exposure, Sprague–Dawley rats were assigned to 3 groups: control, low dose (100 mg/kg body weight), and high dose (500 mg/kg body weight), and exposed to two different sizes of silver nanoparticles (average diameter 10 and 25 nm) over 28 days. Thereafter, the rats were allowed to recover for 4 months. Regardless of the silver nanoparticle size, the silver content in most tissues gradually decreased during the 4-month recovery period, indicating tissue clearance of the accumulated silver. The exceptions were the silver concentrations in the brain and testes, which did not clear well, even after the 4-month recovery period, indicating an obstruction in transporting the accumulated silver out of these tissues. Therefore, the results showed that the size of the silver nanoparticles did not affect their tissue distribution. Furthermore, biological barriers, such as the blood–brain barrier and blood-testis barrier, seemed to play an important role in the silver clearance from these tissues.

## Introduction

Among nanomaterials, the commercial application of silver nanoparticles is the most widespread, where their antimicrobial activity has been applied to bedding, washing machines, water purification, toothpaste, shampoo and rinse, nipples and nursing bottles, fabrics, deodorants, filters, kitchen utensils, toys, and humidifiers [1]. Silver nanoparticles have also been added to medical products, including wound dressings, contraceptives, surgical instruments, bone prostheses, and cardiac catheters [2,3].

In previous research, the target organs for silver nanoparticles have been shown to be the liver in a 28-day oral toxicity study [4] and 90-day oral subchronic study [5], and the liver and lungs in 90-day inhalation studies [6,7]. As a result, these studies set the NOAEL at 30 mg/kg/body weight [4,5] in the 28-day oral toxicity study and 90-day oral subchronic study, and 100 μg/m^3^[6] and 117 μg/m^3^[7] in the 90-day inhalation study and 12-week toxicity study, respectively. Further studies have also found that silver originating from silver nanoparticles is distributed in all tissues, including the liver, kidneys, lungs, spleen, brain, blood, ovaries, and testes [8-10]. A gender-related distribution of silver in the kidneys was also consistent in the results from the abovementioned 28-day, 90-day, and 12-week inhalation studies [4,6,7] and 90-day oral studies [5]. Thus, the clearance behavior is an important determinant for predicting the chronic effects of tissue-accumulated silver nanoparticles. Several studies have already investigated the clearance of tissue-accumulated silver following iv (intravenous) or oral exposure to different sizes of silver nanoparticles and silver ions, for example, following 5 days of iv exposure to 17 days post exposure [8], following 28 days of oral exposure to 8 weeks post exposure [9], and following 90 days of inhalation exposure to 90 days post exposure [7]. However, there has been no long-term post exposure study of the clearance kinetics of tissue-accumulated silver following subacute oral silver nanoparticle exposure. Accordingly, in this study, rats were administered silver nanoparticles orally for 28 days and allowed to recover for four months to identify the clearance of the tissue-accumulated silver.

## Materials and methods

### Silver nanoparticles

The colloidal silver nanoparticles (CAS No. 7440-22-4) were purchased from ABC Nanotech (Daejeon, Korea) and were at least 99.98% pure. The silver nanoparticles were synthesized based on an inductive coupled plasma (ICP) method using a precursor, such as silver wire. The silver nanoparticles were then immediately stabilized using citrate (0.9%). The detailed synthesis process is described in Lee et al. [11]. The percentage of silver ions in the silver nanoparticle preparations was determined by centrifugation through a cellulose filter with a nominal cutoff value of 3 kDa (Ultra-4, Amicon, Millipore, Germany). The total silver content in the unfiltered silver nanoparticle suspensions and their respective filtrates was measured using ICP-MS. The percentage of soluble silver in the silver nanoparticle suspensions was calculated by dividing the silver content in the filtrates by the silver content in the unfiltered silver nanoparticle suspensions multiplied by 100 [9].

### Transmission electron microscopy

The silver nanoparticles in the 0.9% citrate solution were filtered using filters coated with carbon, mounted on an electron microscope grid (200 mesh, Veco, Eerbeek, Holland), and visualized under a transmission electron microscope (TEM, Hitachi 7100). The diameters of 800 (for 10 nm) and 500 (for 25 nm) randomly selected particles were measured at a magnification of 50,000, and the silver particles analyzed using an energy-dispersive x-ray analyzer (EDX-200, Horiba, Japan) at an accelerating voltage of 75 kV.

### Animals and conditions

Four-week-old male and female, specific-pathogen free (SPF) Sprague Dawley rats were purchased from Orient Bio (Korea) and acclimated for 7 days before starting the experiments. During the acclimation and experimental periods, the rats were housed in polycarbonate cages (maximum of 3 rats per cage) in a room with controlled temperature (22.2 ± 1.7°C) and humidity (48.4 ± 6.0%), and a 12-h light/dark cycle. The rats were fed a rodent diet (Harlan Laboratories Inc., USA) and filtered water *ad libitum*. The rats were divided into 3 groups (20 rats in each group): vehicle control (0.9% citrate), low-dose group (100 mg/kg/day), and high-dose group (500 mg/kg/day). After reaching five weeks of age, the rats were exposed to silver nanoparticles (suspended in 0.9% citrate) for 4 weeks (once/day, 7 days/week) via repeated gavage feeding (dosing volumes were 5 ml/kg). The nanoparticles were freshly prepared every day based on the rat body weights and used immediately. The dose levels were selected based on previous observations from a 28-day oral toxicity study by Kim et al. [4]. The administration of silver nanoparticles was ceased after 28 days, and the rats allowed to recover. Animals were sacrificed at the end of the exposure, and 1, 2, and 4 months after ceasing the silver nanoparticle exposure to investigate the clearance of the tissue-accumulated silver. The experiment was approved by the KCL Institutional Animal Care and Use Committee according to the Korean Animal Care Act.

### Biochemical, hematological, and histopathological evaluation

Food was withheld for 24 h before necropsy at the conclusion of the 28-day oral administration and after 1, 2, and 4 months of recovery, and the rats anesthetized with pentoparbital. Blood was then drawn from the abdominal aorta, collected in heparinized vacutainers, and analyzed for ALB (albumin), ALP (alkaline phosphatase), Ca (calcium), CHO (cholesterol), CRE (creatinine), gamma-GT (gamma-glutamyl transpeptidase), GLU (glucose), GOT (glutamic oxalacetic transaminase), GPT (glutamic pyruvic transaminase), IP (inorganic phosphorus), LDH (lactate dehydrogenase), Mg (magnesium), TP (total protein), UA (uric acid), BUN (blood urea nitrogen), TBIL (total bilirubin), CK (creatine phosphokinase), Na (sodium),K(potassium), Cl (chloride), TG (triglyceride), and A/G (ratio of albumin to globulin) using a biochemical blood analyzer (Hitachi 7180, Hitachi, Japan). The WBC (white blood cell count), RBC (red blood cell count), Hb (hemoglobin concentration), HTC (hematocrit), MCV (mean corpuscular volume), MCH (mean corpuscular hemoglobin), MCHC (mean corpuscular hemoglobin concentration), RDW (red cell distribution width), PLT (platelet count), MPV (mean platelet volume), NE# (number of neutrophils), NE% (percent of neutrophils), LY# (number of lymphocytes), LY% (percent of lymphocytes), MO# (number of monocytes), MO% (percent of monocytes), EO# (number of eosinophils), EO% (percent of eosinophils), BA# (number of basophils), and BA% (percent of basophils) were also analyzed using a blood cell counter (Hemavet 0950, CDC Technology, USA). Following the blood collection, the rats were sacrificed by cervical dislocation and the adrenal glands, bladder, ovaries, uterus, epididymis, seminal vesicle, heart, thymus, thyroid gland, trachea, esophagus, tongue, prostate, lungs, nasal cavity, kidneys, spleen, liver, pancreas, and brain all carefully removed. The organs were then weighed, fixed in a 10% formalin solution containing neutral phosphate-buffered saline, embedded in paraffin, stained with hematoxylin and eosin, and finally examined using light microscopy. Meanwhile, the testes were fixed using Bouin’s solution. Two control groups were used with 5 animals in each, however, the results were pooled for the analysis in the Additional file 1.

### Determination of tissue silver

The organs fixed in 10% formaline solutions for the histopathological evaluation were also used for the tissue silver determination. The tissues were digested with concentrated nitric acid using a microwave digestion system (MARS 230/60, CEM). Thereafter, the concentration of silver in the digested fluid was analyzed according to a flameless method using an atomic absorption spectrophotometer equipped with a Zeeman graphite furnace (Perkin Elmer 5100ZL, Zeeman Furnace Module, USA) based on the NIOSH 7300 method [12]. The concentration of silver in the tissues was expressed as the μg/g wet weight. The detection limit was 0.24 ppb and the limit of quantification was 0.797 ppb.

### Silver clearance from tissues during 4-month recovery period

The elimination half-life (t_1/2_) of the silver in the blood and various biological tissues 1, 2, and 4 months following the oral administration of silver nanoparticles was obtained by analyzing the terminal phase of the silver concentration vs. the time profiles using the nonlinear least squares regression software WinNonlin (Pharsight, Cary, NC, USA).

### Statistical analyses

The statistical analyses were performed using SPSS (Version 12). The statistical evaluation included a two-tailed Student’s *t*-test or analysis of variance (ANOVA) following multiple comparison tests using Duncan’s method. The level of statistical significance was set at p < 0.05.

## Results

### Silver nanoparticle characterization

**Figure 1:**
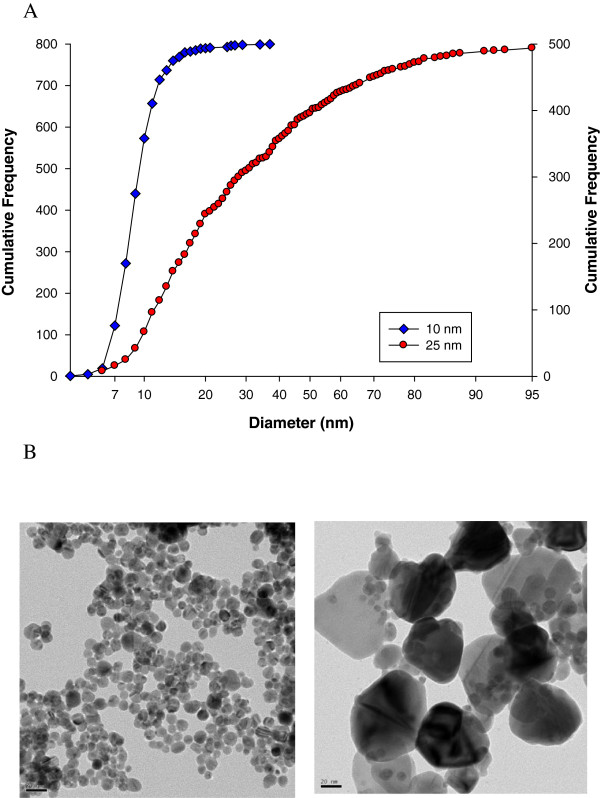
**Particle size distribution and TEM micrographs of silver nanoparticles. A**. Particle size distribution. Blue squares, 10 nm; red circles, 25 nm. **B**. TEM micrographs.

The count median diameter and geometric standard deviation of the silver nanoparticles in the 0.9% citrate solution (Sodium citrate tribasic dihydrate, Sigma USA) analyzed by transmission electron microscopy were 10 nm/1.28 and 25 nm/ 2.1, respectively (Figure 1). The difference in the average size was shown to be significant (P < 0.01) using a Student *t*-test. The percentage of silver ions in the 10 nm and 25 nm silver nanoparticles was 0.0008% and 0.002%, respectively.

### Animal observation, food consumption, and effect on body and organ weights

**Figure 2:**
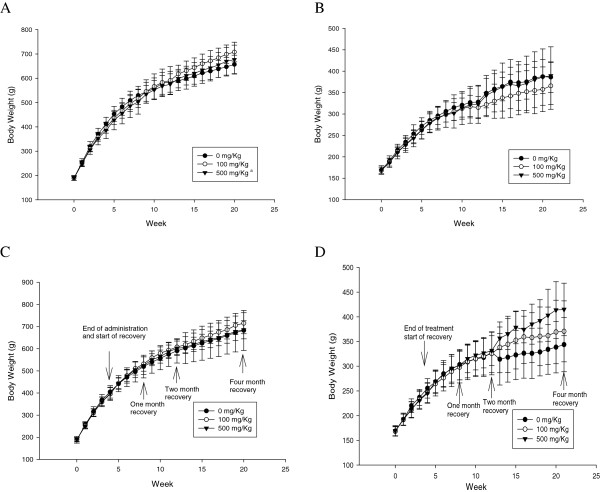
**Body weight changes during 28-day oral administration of silver nanoparticles and 1, 2, and 4 months of recovery.** a, Significant difference vs. control, p < 0.05 (3–4 weeks). **A**. Male (10 nm), 0–4 weeks (n = 20); 5–8 weeks (n = 15); **B**. Female (10 nm), 0–4 weeks (n = 20); 5–8 weeks 9–12 weeks (n = 10); 13–20 weeks (n = 5). (n = 15); 9–12 weeks (n = 10); 13–21 weeks (n = 5). **C**. Male (25 nm), 0–4 weeks (n = 20); 5–8 weeks (n = 15); **D**. Female (25 nm), 0–4 weeks (n = 20); 5–8 weeks 9–12 weeks (n = 10); 13–20 weeks (n = 5). (n = 15); 9–12 weeks (n = 10); 13–21 weeks (n = 5).

There were no significant differences in the food consumption and water intake between the treated male and female rats and the control group (data not shown). Plus, no significant dose-related changes were observed in the male body weights during both the silver nanoparticle treatment and the recovery period (Figure 2). However, the female rats did show body weight differences at the two-month recovery point between the control and the middle-dose group with the 10 nm treatment and between the control and the high and middle-dose groups with the 25 nm treatment, yet none of these dose-related changes was statistically significant. There were no signs of infection or adverse effects during the recovery period.

### Biochemical, hematological, and histopathological evaluation

The histopathology examinations after 28 days administration of 10 nm or 25 nm silver nanoparticles and after one or two months of recovery did not reveal any dose-dependent lesions in the liver (Additional file 1: Tables S1-S6). While the 28-day treatment group and one and two-month recovery groups did show lipid droplets and inflammatory cell infiltration in the liver and tubular regeneration in the kidneys, these observations were not dose-dependent (Additional file 1: Table S7).

The hematological parameters did not show any significant changes after 28 days of silver nanoparticle administration or after one or two months of recovery, except for a decreased MCHC (mean corpuscular hemoglobin concentration) in the high-dose female rats (25 nm) from the one and two-month recovery groups (Additional file 1: Tables S12 & S13) and a decreased platelet count in the high-dose female rats (25 nm) from the two-month recovery group (Additional file 1: Table S13). The prothrombin time (PT) was significantly increased (P < 0.05) in the high-dose (25 nm) female rats when compared with the control (Additional file 1: Table S14). However, the PT and APPT (active partial thromboplastin time) did not show any significant change in either the male or female rats from the one and two-month recovery groups (Additional file 1: Tables S15 & S16).

When compared with the control group, the cholesterol (CHOL) level was significantly higher (P < 0.05) in the male rats after being treated with the low or high dose of 10 nm silver nanoparticles and the low dose of 25 nm silver nanoparticles for 28 days (Additional file 1: Table S17). However, this increase returned to a normal level after one or two months of recovery (Additional file 1: Tables S18 & S19). The level of inorganic phosphorus was significantly higher (P < 0.01) in the high-dose (25 nm) male rats after 2 months of recovery (Additional file 1: Table S19). The level of alkaline phosphatase (ALP) was significantly higher (P < 0.01) in the female rats treated with the low or high dose of silver nanoparticles (10 nm) for 28 days (Additional file 1: Table S20). The level of aspartate aminotransferase (AST) was significantly higher (P < 0.05) in the high-dose (25 nm) groups (Additional file 1: Table S20). In addition, the ALP level was significantly higher (P < 0.01) in the low-dose (10 nm) female rats after one month of recovery (Additional file 1: Table S21). However, the AST and ALP levels in the female rats all returned to normal after 2 months of recovery (Additional file 1: Table S22). Some of the increases in the hepatotoxicity-relevant markers (CHOL, ALP, and AST) following the silver nanoparticle exposure were consistent with previous observations related to the subacute and subchronic oral toxicity of silver nanoparticles. Yet, most of the hepatotoxicity marker levels returned to normal after 2 months of recovery.

3.4. Silver concentrations in blood, liver, kidneys, spleen, testes, ovaries, and brain after 4 months of recovery following silver nanoparticle administration.

**Figure 3:**
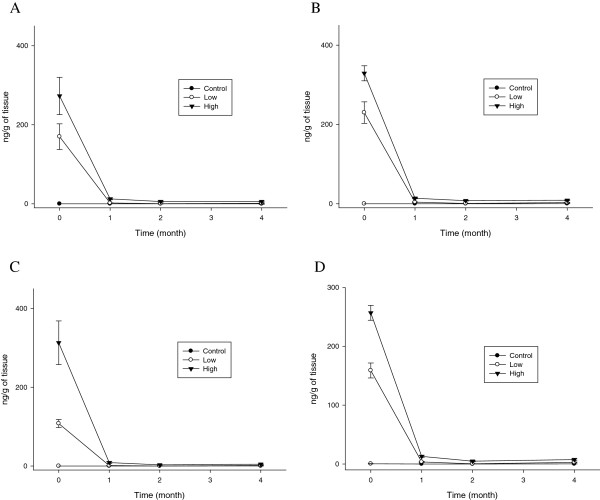
**Blood silver concentrations (*n*g/g tissue wet weight) before and after cessation of silver nanoparticle administration.** The rats were allowed to recover for 1, 2, and 4 months following 28 days of oral silver nanoparticle exposure. **A**. Male (10 nm). **B**. Female (10 nm). **C**. Male (25 nm). **D**. Female (25 nm).

The blood silver concentrations rapidly decreased during the first month following the cessation of the silver nanoparticle administration and then maintained these levels for the remainder of the 4- month recovery period (Figure 3). For the 10 nm silver nanoparticles, the blood silver t_1/2_ in the high-dose group was 98.94 and 78.14 days for the male and female rats, respectively. However, for the 25 nm silver nanoparticles, the blood silver t_1/2_ in the high-dose group was longer at 133.37 and 140.12 days for the male and female rats, respectively (Table 1).

**Table 1:** **Clearance T**_**1/2 **_**of silver after 28 days of oral administration (superscript 0, 0-order; 1, 1st-order)**

**Tissue**	**Male**				**Female**			
	**100 mg/kg/day**		**500 mg/kg/day**		**100 mg/kg/day**		**500 mg/kg/day**	
	**Half life (days)**	**R square**	**Half life (days)**	**R square**	**Half life (days)**	**R square**	**Half life (days)**	**R square**
A. 10 nm								
Blood^0^	76.88	0.47	98.94	0.58	77.33	0.47	78.14	0.48
Brain^1^	260.78	0.96	95.98	0.93	140.23	1.00	77.97	0.88
Kidneys^1^	29.75	0.94	69.81	0.91	79.60	0.71	77.49	0.89
Liver^1^	38.58	0.81	24.05	1.00	189.29	0.09	74.70	0.90
Spleen^1^	48.67	1.00	54.98	0.98	56.11	0.85	56.04	0.89
Testes/ovaries^1^	55.03	1.00	N/A	N/A	34.79	0.95	35.77	0.92
B. 25 nm								
Blood^0^	77.52	0.47	133.27	0.48	141.45	0.47	140.12	0.48
Brain^1^	136.03	0.77	81.06	0.96	124.08	1.00	60.05	0.90
Kidneys^1^	32.07	1.00	53.70	0.99	45.60	0.97	87.87	0.82
Liver^0^	70.10	0.70	62.94	0.82	60.72	0.66	68.29	0.49
Spleen^1^	30.93	0.99	164.91	0.74	66.32	0.93	66.97	0.94
Testes/ovaries^1^	64.59	1.00	N/A	N/A	61.85	0.84	33.91	0.95

N/A not applicable (Clearance T_1/2_ not obtainable).

**Figure 4:**
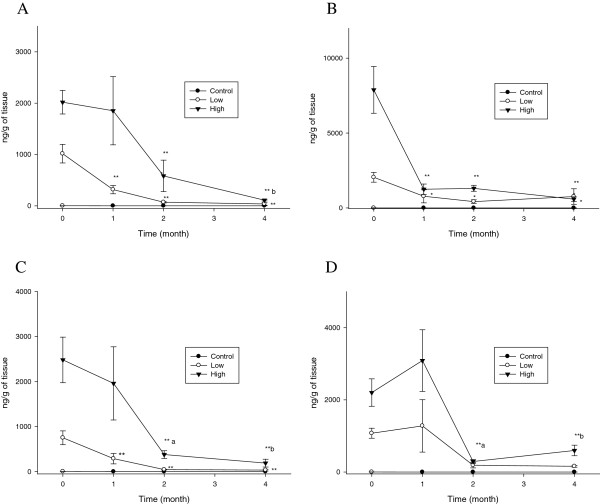
**Liver silver concentrations (*n*g/g tissue wet weight) before and after cessation of silver nanoparticle administration.** The rats were allowed to recover for 1, 2, and 4 months following 28 days of oral silver nanoparticle exposure. **, P < 0.01 28-day exposure vs. recovery; *, P < 0.05 28-day exposure vs. recovery; a, P < 0.05 1-month recovery vs. 2-month recovery; b, P < 0.05 1-month recovery vs. 4-month recovery. **A**. Male (10 nm). **B**. Female (10 nm). **C**. Male (25 nm). **D**. Female (25 nm).

Meanwhile, the liver silver concentrations continually decreased over the 4-month period, showing a rapid decrease during 2 months following the cessation of the silver nanoparticle administration (P < 0.05-0.01) for the high-dose male and female rats treated with either the 10 nm or 25 nm silver nanoparticles, and the low-dose female rats treated with the 25 nm silver nanoparticles (Figure 4). For the male rats, the liver silver t_1/2_ was slightly longer for the 25 nm silver nanoparticles than for the 10 nm silver nanoparticles, however, this was not the case for the female rats treated with the 10 nm silver nanoparticles (Table 1).

**Figure 5:**
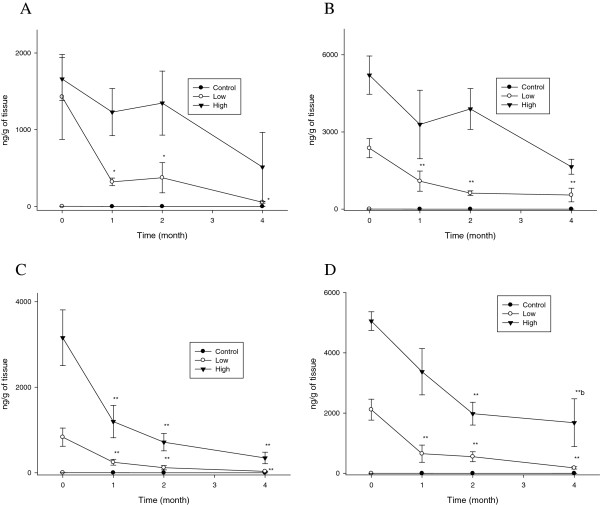
**Kidney silver concentrations (*n*g/g tissue wet weight) before and after cessation of silver nanoparticle administration.** The rats were allowed to recover for 1, 2, and 4 months following 28 days of oral silver nanoparticle exposure. **, P < 0.01 28-day exposure vs. recovery; *, P < 0.05 28-day exposure vs. recovery; a, P < 0.05 1-month recovery vs. 2-month recovery; b, P < 0.05 1-month recovery vs. 4-month recovery. **A**. Male (10 nm). **B**. Female (10 nm). **C**. Male (25 nm). **D**. Female (25 nm).

The kidney silver content persisted for 4 months after cessation in the high-dose male and female rats treated with the 10 nm silver nanoparticles, whereas the silver content decreased significantly (P < 0.05-0.01) after 1 month in the low-dose male and female rats treated with the 10 nm silver nanoparticles (Figure 5). For the rats treated with the 25 nm silver nanoparticles, the kidney silver content decreased significantly (P < 0.01) after 1 month in both the low and high-dose male and female rats, with the exception of the high-dose female rats that only showed a significant decrease after 2 months (Figure 5). The kidney silver t_1/2_ in the high-dose male rats was slightly longer for the 10 nm silver nanoparticles than for the 25 nm silver nanoparticles, while overall the kidney silver t_1/2_ was slightly longer in the female rats than in the male rats for both the 10 and 25 nm silver nanoparticles (Table 1).

**Figure 6:**
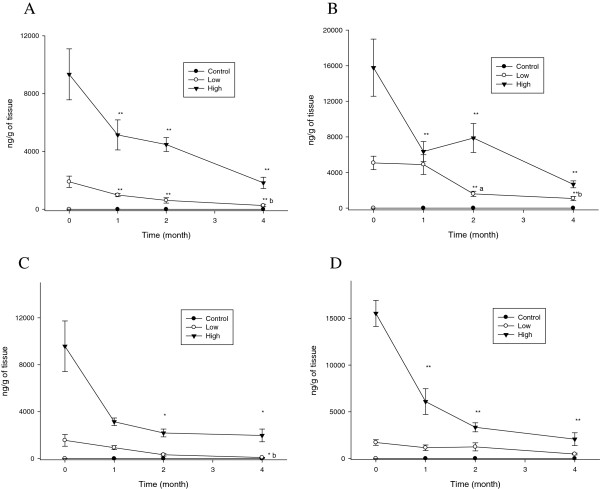
**Spleen silver concentrations (*n*/g tissue wet weight) before and after cessation of silver nanoparticle administration.** The rats were allowed to recover for 1, 2, and 4 months following 28 days of oral silver nanoparticle exposure. **, P < 0.01 28-day exposure vs. recovery; *, P < 0.05 28-day exposure vs. recovery; a, P < 0.05 1-month recovery vs. 2-month recovery; b, P < 0.05 1-month recovery vs. 4-month recovery. **A**. Male (10 nm). **B**. Female (10 nm). **C**. Male (25 nm). **D**. Female (25 nm).

The spleen silver concentration also showed a significant (P < 0.01) decrease after 1 month in the high-dose male and female rats treated with either the 10 nm or 25 nm silver nanoparticles, whereas the low-dose male and female rats treated with the 10 nm or 25 nm silver nanoparticles showed a relatively slower clearance (Figure 6). The spleen silver t_1/2_ was slightly longer for the 25 nm silver nanoparticles than for the 10 nm silver nanoparticles, except in the case of the low-dose male rats treated with the 25 nm silver nanoparticles (Table 1).

**Figure 7:**
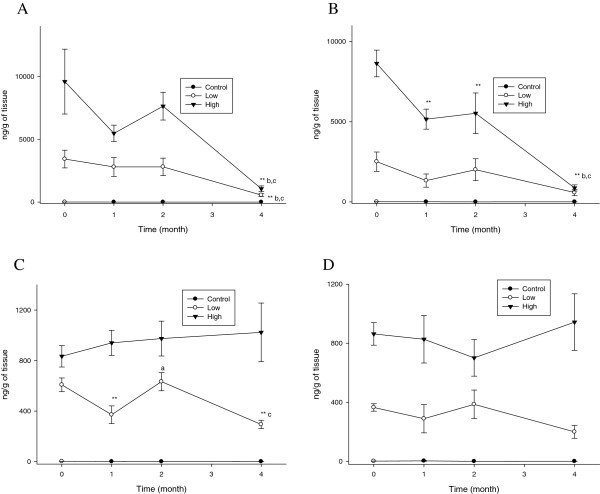
**Ovary and testis silver concentrations (*n*g/g tissue wet weight) before and after cessation of silver nanoparticle administration.** The rats were allowed to recover for 1, 2, and 4 months following 28 days of oral silver nanoparticle exposure. **, P < 0.01 28-day exposure vs. recovery; *, P < 0.05 28-day exposure vs. recovery; b, P < 0.05 1-month recovery vs. 4-month recovery; c P < 0.05 2-month recovery vs. 4-month recovery. **A**. Ovaries (10 nm). **B**. Ovaries (25 nm). **C**. Testes (10 nm). **D**. Testes (25 nm).

The ovaries showed a rather longer clearance in the low and high-dose female rats treated with the 10 nm silver nanoparticles, only showing a significant (P < 0.01) decrease after 4 months of recovery (Figure 7). In contrast, the ovaries in the high-dose female rats treated with the 25 nm silver nanoparticles showed a significant (P < 0.01) decrease after 1 month of recovery, whereas the ovaries in the low-dose female rats treated with the 25 nm silver nanoparticles did not show any significant decrease during the recovery period. The ovary silver t_1/2_ was similar in the low and high-dose female rats treated with the 10 nm silver nanoparticles, yet longer than the ovary silver t_1/2_ in the low-dose female rats treated with the 25 nm silver nanoparticles (Table 1).

The silver concentrations in the testes did not show any decrease in the male rats treated with either the 10 nm or 25 nm silver nanoparticles, with the exception of the low-dose male rats treated with the 10 nm silver nanoparticles that showed a significant (P < 0.01) decrease after 4 months of recovery (Figure 7). The testis silver t_1/2_ was 55.03 days or 64.59 days for the low-dose male rats treated with the 10 nm or 25 nm silver nanoparticles, respectively (Table 1). The testis silver t_1/2_ was not applicable to the high-dose male rats treated with the 10 or 25 nm silver nanoparticles (Table 1).

**Figure 8:**
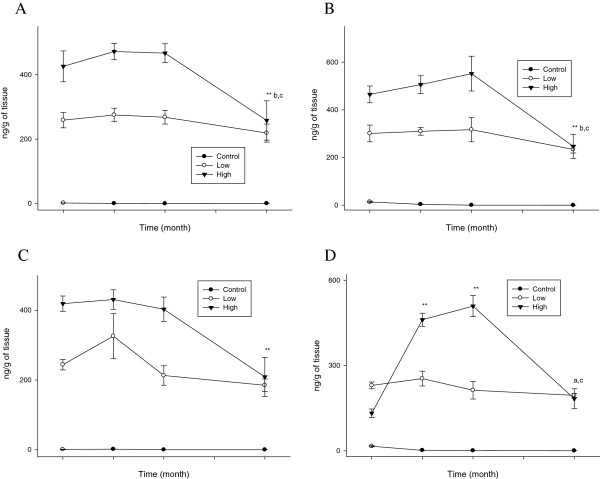
**Brain silver concentrations (*n*g/g tissue wet weight) before and after cessation of silver nanoparticle administration.** The rats were allowed to recover for 1, 2, and 4 months following 28 days of oral silver nanoparticle exposure. **, P < 0.01 28-day exposure vs. recovery; *, P < 0.05 28-day exposure vs. recovery; b, P < 0.05 1-month recovery vs. 4-month recovery; c P < 0.05 2-month recovery vs. 4-month recovery. **A**. Male (10 nm). **B**. Female (10 nm). **C**. Male (25 nm). **D**. Female (25 nm).

The brain also showed difficulty clearing the accumulated silver after a prolonged period of recovery. For the male and female rats treated with the 10 nm or 25 nm silver nanoparticles, the high-dose male and female rats showed a significant decrease (P < 0.01) after 4 months of recovery, except for the high-dose female rats treated with the 25 nm silver nanoparticles that showed a significant decrease (P < 0.01) after 1–2 months of recovery, whereas the low-dose male and female rats treated with the 10 nm or 25 nm silver nanoparticles did not show any clearance (Figure 8). The brain silver t_1/2_ in the low-dose male and female rats was longer than that in the high-dose male and female rats treated with either the 10 nm or 25 nm silver nanoparticles, plus the brain silver t_1/2_ for the 10 nm silver nanoparticles was longer than that for the 25 nm silver nanoparticles (Table 1).

The silver biopersistence in various tissues was further evaluated by an independent elimination rate parameter, the mean residence time (MRT), which represents the mean time the drug molecules reside in the body and is calculated based on the ratio of AUMC_0-tlast_/AUC_0-tlast_, where AUMC is the area of concentration × time vs. a time curve, and AUC is the area of concentration vs. a time curve. As shown in Table 2, the silver MRTs for the brain and testes/ovaries were longer than those for the other tissues, suggesting a persistent accumulation of silver in the brain and testes/ovaries. Also, the particle size had no significant effect on the MRT.

**Table 2:** Mean residence time (MRT) of silver after 28 days of oral administration

**Tissue**	**Male**		**Female**	
	**100 mg/kg/day**	**500 mg/kg/day**	**100 mg/kg/day**	**500 mg/kg/day**
	**(days)**	**(days)**	**(days)**	**(days)**
A. 10 nm				
Blood	2.85	10.02	4.70	11.24
Brain	58.60	54.44	57.82	53.54
Kidneys	31.03	49.12	42.10	49.60
Liver	21.49	32.05	48.77	29.82
Spleen	38.24	43.43	39.52	43.58
Testes/Ovaries	55.32	63.68	44.70	44.19
B. 25 nm				
Blood	3.75	6.47	5.53	11.25
Brain	55.06	52.43	58.57	56.93
Kidneys	25.80	34.67	34.33	46.72
Liver	22.40	29.99	33.89	37.42
Spleen	30.75	40.95	48.11	35.81
Testes/Ovaries	55.34	63.31	48.29	41.75

## Discussion

The objective of this study was to evaluate the silver clearance from tissues following the cessation of silver nanoparticle administration. The silver concentrations in the blood rapidly decreased during the first month of recovery and continued until 4 months, indicating continuous partitioning from the tissues to the blood. Other tissues, including the liver, spleen, ovaries, and kidneys, also showed a degree of clearance of the accumulated silver during the 4-month recovery period. However, the silver concentrations in the testes and brain did not decrease to the control levels, even after the 4-month recovery period, indicating that silver clearance is difficult across biological barriers, such as the blood–brain barrier or blood-testis barrier. The silver half-life in each tissue type was calculated using a zero-order elimination model (Table 1). However, since the R^2^ value was not significantly improved when compared with that calculated using a first-order elimination model, the zero-order elimination was only applied to the blood and liver treated with the 25 nm silver nanoparticles. A half-life estimation based on a zero-order elimination is primarily dependent on the initial concentration change. In previous literature, the systemic disposition of silver has also been described using first-order inter-compartment rate constants [8]. Yet, a large inter-subject variability due to destructive sampling can result in a poor half-life estimation, and inter-individual variations of silver concentrations in biological samples are already known to be very high. Thus, the MRT, which represents the mean residence time of silver nanoparticles in tissue, would seem to be a better indicator of silver biopersistence. In the present study, the MRT showed similar values, regardless of the dose level, thereby indicating the biopersistence more clearly than the t_1/2_ values.

Although the clearance half-times differed according to dose and gender, the tissues with no biological barrier, such as the liver, kidneys, and spleen, showed a similar clearance trend. Furthermore, the MRT also showed differences in the silver biopersistence in various tissues. Thus, the silver concentration clearance was in the order of blood > liver = kidneys > spleen > ovaries > testes = brain. Therefore, the silver clearance from tissues containing biological barriers would appear to be differently regulated. As a result, the silver biopersistence in the testes and brain can complicate the risk assessment of silver nanoparticles. Silver biopersistence in the brain and testes was already observed in the case of 28 days of oral administration of silver nitrate (9 mg/kg bw/day), noncoated (<20 nm), and PVP-coated silver nanoparticles (90 mg/kg bw/day) followed by 8 weeks of recovery [9]. However, the short-term (5 days) intravenous injection of various sizes (20, 80, and 110 nm) of silver nanoparticles followed by 17 days of recovery did not reveal any silver biopersistence in the brain and testes [8].

Wijnhoven et al., [13] previously hypothesized that the toxic effect of silver is proportional to the free silver ions, yet how this relates to silver nanoparticles remains unclear. Notwithstanding, the results would seem to indicate two possible ways of clearing accumulated silver from the body: as silver ions or silver nanoparticles. However, there are clear difficulties in evaluating the silver ions originating from silver nanoparticles *in vivo* and estimating the fraction of ionization from silver nanoparticle surfaces in various tissues.

In the present study, the silver nanoparticles were dispersed in citrate after gas-phase synthesis, as described by Lee et al. [11]. Stabilizing the layers prevents silver nanoparticle aggregation, enabling the nanoparticles to remain suspended in the water column. Silver nanoparticles are normally stabilized using a charged surface coating, such as citrate, that creates an electrostatic barrier to aggregation. In a natural environment, a stabilizing agent such as citrate, which is weakly complexed with the silver nanoparticle surfaces, can be rapidly displaced by ligands, such as sulfides [14]. Surface-bound ligands, such as citrate, compete with oxygen for surface sites and thus decrease the rate of oxidation. Liu et al. provided evidence of this mechanism by demonstrating a decrease in the Ag + released from silver nanoparticles as the citrate surface coatings became more densely-packed [15]. Nanoparticles in the GI tract show a different behavior depending on the pH in the stomach (pH 2–3) and intestines (pH 7.8), where unstable and agglomerated nanoparticles disperse well in an acidic and basic pH [16]. Liu et al. recently proposed a conceptual model of ingested silver nanoparticles in the human body. In their model, argyrial silver deposits are not translocated engineered Ag-NPs, but rather secondary particles formed by partial dissolution in the GI tract followed by ion uptake, systemic circulation as organo-Ag complexes, and immobilization as zerovalent Ag-NPs by photoreduction in light-affected skin regions [17]. The secondary Ag-NPs then undergo detoxifying transformations into sulfides and further into selenides or Se/S mixed phases through exchange reactions. The formation of secondary particles in biological environments implies that Ag-NPs are not only a product of industrial nanotechnology, but have also been long present in the human body following exposure to more traditional chemical forms of silver. Therefore, the lack of any difference in tissue distribution between the 10 and 25 nm silver nanoparticles after 28 days of oral administration indicated that the ingested silver nanoparticles were dissolved in the low pH gastric fluid environment, which then led to silver ion release. The silver ions and their complexes are brought into the bloodstream through ion or nutrient uptake channels and circulate systematically. Plus, the majority of silver in the circulation is predicted to be bound to thiol complexes, which have high binding affinities yet are easily exchangeable, giving Ag(I) a significant biomolecular mobility [17]. Similarly, *in vivo* nanoparticle formation from silver ions has also been suggested following the detection of silver-containing nanoparticles in several tissues obtained from animals administered silver nitrate or silver nanoparticles orally for 28 days [9]. Yet, the uptake of silver nanoparticles through the intestines cannot be excluded. In a previous 90-day silver nanoparticle (average 56 nm) oral administration study, a dose-dependent increase in the villi pigmentation was observed (presumably yet not confirmed as silver nanoparticle related) [5].

The formaline solution-based tissue preparation used in the present study did not allow the nanoparticles in the tissue samples to be examined by TEM. Yet, while TEM can be used to visualize silver nanoparticles in tissues, the particle shapes of the silver nanoparticles cannot be confirmed without the use of additional defining methodologies, such as EDX (energy dispersive X-ray analyzer). And even a TEM-EDX analysis may not provide exact composition information on target particles. Furthermore, the tissue presence of secondary particles generated from silver-ion complexes can also complicate whether particles are silver nanoparticles or secondary silver ion complexes. Thus, other recent technologies, such as hyperspectral images, need to be used in future studies.

In previous studies, certain forms of Mn (Manganese) have been reported to affect the brain. For example, in the case of rats, inhalation exposure to soluble forms of Mn, such as Mn sulfate and Mn phosphate, was found to increase the brain Mn concentration when compared to inhalation exposure to the less soluble Mn tetraoxide [18]. After the cessation of welding-fume exposure (average diameter 100 nm), the absence of any further supply of soluble Mn to the lungs resulted in a reduction of the Mn concentrations in the blood and other tissues, including the brain, liver, and spleen [19]. However, unlike Mn accumulation in the brain, the silver in this study exhibited a different pattern, showing poor clearance from brain tissue.

Furthermore, the different nanoparticle sizes used in this study did not have much effect on the ADME (absorption, distribution, metabolism, and excretion), although the size difference was relatively narrow. The distribution and clearance of silver from various tissues showed a similar pattern. Similarly, in other studies, the inhalation of 18–19 nm [6] and 12–15 nm [20] silver nanoparticles and oral dosing with 56–60 nm silver nanoparticles [4,5] showed a similar tissue distribution. Therefore, this would seem to confirm the applicability of the 0-hypothesis for silver suggested by Wijnhoven et al. [18] and chemical transformation suggested by Lie et al. [17], which state that silver toxicity mainly originates from silver ions, in this case generated from the surface of the silver nanoparticles.

A certain level of liver toxicity was indicated by the increase of cholesterol (CHOL) in the male rats following the 28-day oral administration of the low and high dose of 10 nm silver nanoparticles and low dose of 25 nm silver nanoparticles, the increase of alkaline phosphatase (ALP) in the female rats following the low and high dose of 10 nm silver nanoparticles, and the increase of aspartate aminotransferase (AST) in the female rats following the high dose of 25 nm silver nanoparticles. While one case of bile duct hyperplasia was observed among the male rats treated with the 10 nm silver nanoparticles, the percent of inflammatory cell infiltration in the male rats was only mildly increased by the 10 nm and 25 nm silver nanoparticle treatments. The present study did not observe the more prominent bile duct hyperplasia reported in a previous 28-day oral study [4] and 90-day oral study [5]. Thus, the development of bile duct hyperplasia may require a longer exposure duration than 28 days.

The present study also found that coating the silver nanoparticles, in this case with citrate, did not have any effect on the ADME when compared with the use of un-coated silver nanoparticles, including silver nanoparticles generated by evaporation/condensation, as used in other inhalation toxicity studies [6,17], dry powder, as used in other oral toxicity studies [4,5], and PVP-coated silver nanoparticles [9]. When evaluating the toxicity of the citrate-coated silver nanoparticles used in this study, the extent of the toxicity was quite similar to that of the dry powder used in the previous studies by Kim et al. [4,5].

Thus, when including silver biopersistence, especially in the brain and testes, this complicates the risk assessment of silver nanoparticles. Therefore, the NOAELs previously reported by Kim et al. [5] based on a subchronic oral toxicity study and Sung et al. [6] based on a subchronic inhalation toxicity study need to be adjusted in the light of biopersistence. Until now, histopathological evaluations of the testes and brain have not show any apparent toxicity, consequently, new tools are needed to evaluate biopersistent-relevant toxicity. Nonetheless, the current findings of minimized effects from silver nanoparticle coating and size differences on the ADME could simplify the risk assessment of silver nanoparticles.

## Competing interest

The authors report no conflicts of interest. The authors alone are responsible for the content and writing of this article.

## Authors’ contributions

IJY headed the study and performed the pathology and data analysis together with YSK. YSK, KSS, HRR, and JHS conducted the animal experiments. JHL, DM, and JDK analyzed the silver content in the animal tissues and contributed to the tissue distribution study. HMP, NWS, and KA contributed to the silver nanoparticle characterization. BSS contributed to the toxicokinetic analysis. JHL and IJY conceived and designed the study. All the authors reviewed and interpreted the data, and read and approved the final manuscript.

## Supplementary Material

Additional file 1 Histopathological findings for male and female rats after 28-day oral administration of silver nanoparticles, 1 and 2 month recovery (Tables S1-7); Hematological values for male and female rats after 28 day exposure, 1 and 2 month recovery (Tables S8-13); Plasma coagulation values for male and female rats after 28-day exposure, 1 and 2 month recover (Tables S14-16); Serum biochemical values for male and female rats after 28-day exposure, 1 and 2 month recovery (Tables S17-22); Number of animal used for biochemical and hematological assays (Table S23); Number of animal used for tissue content of silver analysis (Table S24). Click here for file
