# Supplementary material for: Biopersistence of silver nanoparticles in tissues from Sprague–Dawley rats
Source: Part Fibre Toxicol. 2013 Aug 1;10:36. doi: 10.1186/1743-8977-10-36 (PMC3734196; doi:10.1186/1743-8977-10-36)
Supplement: Additional file 1 — Histopathological findings for male and female rats after 28-day oral administration of silver nanoparticles, 1 and 2 month recovery (Tables S1-7); Hematological values for male and female rats after 28 day exposure, 1 and 2 month recovery (Tables S8-13); Plasma coagulation values for male and female rats after 28-day exposure, 1 and 2 month recover (Tables S14-16); Serum biochemical values for male and female rats after 28-day exposure, 1 and 2 month recovery (Tables S17-22); Number of animal used for biochemical and hematological assays (Table S23); Number of animal used for tissue content of silver analysis (Table S24). [file 1743-8977-10-36-S1.doc]

Supplement 1. Histopathological findings for male rats after 28-day oral administration of silver nanoparticles. NSL: No significant lesions found

| GROUP:  DOSE (mg/kg/day): | | Control  0 | | 10 nm  100 | | 10 nm  500 | | 25 nm  100 | | 25 nm  500 | |
| --- | --- | --- | --- | --- | --- | --- | --- | --- | --- | --- | --- |
| Number of Animals | | 10 | | 5 | | 5 | | 5 | | 5 | |
|  |  | N | % | N | % | N | % | N | % | N | % |
| Brain (Cerebrum, Cerebellum) | NSL | 10/10 | 100 | 5/5 | 100 | 5/5 | 100 | 5/5 | 100 | 5/5 | 100 |
| Lungs (Trachea) | NSL | 7/10 | 70 | 4/5 | 80 | 4/5 | 80 | 2/5 | 40 | 2/5 | 40 |
|  | Inflammatory cell infiltration | 3/10 | 30 | 1/5 | 20 | 1/5 | 20 | 1/5 | 20 | 3/5 | 60 |
|  | Hypertropy | 0/10 | 0 | 0/5 | 0 | 0/5 | 0 | 2/5 | 40 | 0/5 | 0 |
| Spleen | NSL | 7/10 | 70 | 5/5 | 100 | 3/5 | 60 | 4/5 | 80 | 4/5 | 80 |
|  | Inflammatory cell infiltration | 1/10 | 10 | 0/5 | 0 | 0/5 | 0 | 0/5 | 0 | 0/5 | 0 |
|  | Degranulation | 2/10 | 20 | 0/5 | 0 | 0/5 | 0 | 1/5 | 20 | 1/5 | 20 |
|  | Hypertropy | 0/10 | 0 | 0/5 | 0 | 2/5 | 40 | 0/5 | 0 | 0/5 | 0 |
| Liver | NSL | 1/10 | 10 | 3/5 | 60 | 0/5 | 0 | 2/5 | 40 | 0/5 | 0 |
|  | Kuffer cells, Increase | 1/10 | 10 | 0/5 | 0 | 0/5 | 0 | 0/5 | 0 | 0/5 | 0 |
|  | Lipid droplets, Diffuse | 5/10 | 50 | 0/5 | 0 | 3/5 | 60 | 3/5 | 60 | 3/5 | 60 |
|  | Inflammatory cell infiltration | 6/10 | 60 | 1/5 | 20 | 5/5 | 100 | 3/5 | 60 | 5/5 | 100 |
|  | Focal, Necrosis | 0/10 | 0 | 1/5 | 20 | 0/5 | 0 | 0/5 | 0 | 0/5 | 0 |
|  | Bile duct, Hyperplasia | 0/10 | 0 | 0/5 | 0 | 1/5 | 20 | 0/5 | 0 | 0/5 | 0 |
| Kidneys | NSL | 3/10 | 30 | 1/5 | 20 | 1/5 | 20 | 1/5 | 20 | 3/5 | 60 |
|  | Inflammatory cell infiltration | 7/10 | 70 | 4/5 | 80 | 4/5 | 80 | 4/5 | 80 | 2/5 | 40 |
|  | Regeneration, Tubules | 0/10 | 0 | 0/5 | 0 | 0/5 | 0 | 0/5 | 0 | 1/5 | 20 |
| Testes | NSL | 9/10 | 90 | 5/5 | 100 | 5/5 | 100 | 5/5 | 100 | 5/5 | 100 |
|  | Organ omission | 1/10 | 10 | 0/5 | 0 | 0/5 | 0 | 0/5 | 0 | 0/5 | 0 |
| Epididymis | NSL | 9/10 | 90 | 5/5 | 100 | 2/5 | 40 | 5/5 | 100 | 5/5 | 100 |
|  | Organ omission | 1/10 | 10 | 0/5 | 0 | 0/5 | 0 | 0/5 | 0 | 0/5 | 0 |
|  | Inflammatory cell infiltration | 0/10 | 0 | 0/5 | 0 | 3/5 | 60 | 0/5 | 0 | 0/5 | 0 |

Supplement 2. Histopathological findings for male rats after 1 month of recovery following 28-day oral administration of silver nanoparticles. NSL: No significant lesions found

| GROUP:  DOSE (mg/kg/day): | | Control  0 | | 10 nm  100 | | 10 nm  500 | | 25 nm  100 | | 25 nm  500 | |
| --- | --- | --- | --- | --- | --- | --- | --- | --- | --- | --- | --- |
| Number of Animals | | 10 | | 5 | | 5 | | 5 | | 5 | |
|  |  | N | % | N | % | N | % | N | % | N | % |
| Brain (Cerebrum, Cerebellum) | NSL | 9/10 | 90 | 5/5 | 100 | 5/5 | 100 | 5/5 | 100 | 5/5 | 100 |
|  | Inflammatory cell infiltration | 1/10 | 10 | 0/5 | 0 | 0/5 | 0 | 0/5 | 0 | 0/5 | 0 |
| Lungs (Trachea) | NSL | 9/10 | 90 | 5/5 | 100 | 5/5 | 100 | 5/5 | 100 | 1/5 | 20 |
|  | Inflammatory cell infiltration | 1/10 | 10 | 0/5 | 0 | 0/5 | 0 | 0/5 | 0 | 4/5 | 80 |
| Spleen | NSL | 8/10 | 80 | 3/5 | 60 | 4/5 | 80 | 2/5 | 40 | 4/5 | 80 |
|  | Atropy | 2/10 | 20 | 0/5 | 0 | 0/5 | 0 | 2/5 | 40 | 0/5 | 0 |
|  | Degranulation | 0/10 | 0 | 1/5 | 20 | 0/5 | 0 | 1/5 | 20 | 1/5 | 20 |
|  | Hypertropy | 0/10 | 0 | 1/5 | 20 | 1/5 | 20 | 0/5 | 0 | 0/5 | 0 |
| Liver | NSL | 0/10 | 0 | 0/5 | 0 | 0/5 | 0 | 0/5 | 0 | 0/5 | 0 |
|  | Lipid droplets, Diffuse | 6/10 | 60 | 3/5 | 60 | 2/5 | 40 | 5/5 | 100 | 2/5 | 40 |
|  | Inflammatory cell infiltration | 10/10 | 100 | 5/5 | 100 | 5/5 | 100 | 5/5 | 100 | 5/5 | 100 |
|  | Focal, Necrosis | 1/10 | 10 | 1/5 | 20 | 0/5 | 0 | 0/5 | 0 | 0/5 | 0 |
| Kidneys | NSL | 5/10 | 50 | 2/5 | 40 | 3/5 | 60 | 1/5 | 20 | 1/5 | 20 |
|  | Inflammatory cell infiltration | 5/10 | 50 | 2/5 | 40 | 2/5 | 40 | 4/5 | 80 | 2/5 | 40 |
|  | Regeneration, Tubules | 1/10 | 10 | 0/5 | 0 | 0/5 | 0 | 1/5 | 20 | 2/5 | 40 |
|  | Cystic change, Medulla | 0/10 | 0 | 1/5 | 20 | 0/5 | 0 | 0/5 | 0 | 0/5 | 0 |
| Testes | NSL | 10/10 | 100 | 5/5 | 100 | 5/5 | 100 | 5/5 | 100 | 5/5 | 100 |
| Epididymis | NSL | 9/10 | 90 | 3/5 | 60 | 4/5 | 80 | 3/5 | 60 | 4/5 | 80 |
|  | Inflammatory cell infiltration | 1/10 | 10 | 2/5 | 40 | 1/5 | 20 | 2/5 | 40 | 1/5 | 20 |

Supplement 3. Histopathological findings for male rats after 2 months of recovery following 28-day oral administration of silver nanoparticles. NSL: No significant lesions found

| GROUP:  DOSE (mg/kg/day): | | Control  0 | | 10 nm  100 | | 10 nm  500 | | 25 nm  100 | | 25 nm  500 | |
| --- | --- | --- | --- | --- | --- | --- | --- | --- | --- | --- | --- |
| Number of Animals | | 10 | | 5 | | 5 | | 5 | | 5 | |
|  |  | N | % | N | % | N | % | N | % | N | % |
| Brain (Cerebrum, Cerebellum) | NSL | 10/10 | 100 | 5/5 | 100 | 5/5 | 100 | 5/5 | 100 | 5/5 | 100 |
| Lungs (Trachea) | NSL | 2/10 | 10 | 0/5 | 0 | 1/5 | 20 | 2/5 | 40 | 1/5 | 20 |
|  | Inflammatory cell infiltration | 7/10 | 70 | 3/5 | 60 | 2/5 | 40 | 3/5 | 60 | 4/5 | 80 |
|  | Osseus clots | 1/10 | 10 | 0/5 | 0 | 0/5 | 0 | 0/5 | 0 | 1/5 | 20 |
|  | Epithelium Metaplasia | 1/10 | 10 | 0/5 | 0 | 0/5 | 0 | 0/5 | 0 | 0/5 | 0 |
|  | Alveolar wall hypertrophy | 0/10 | 0 | 1/5 | 20 | 0/5 | 0 | 0/5 | 0 | 0/5 | 0 |
|  | Bronchiole, Hypertropy | 0/10 | 0 | 1/5 | 20 | 4/5 | 80 | 0/5 | 0 | 0/5 | 0 |
| Spleen | NSL | 8/10 | 80 | 5/5 | 100 | 4/5 | 80 | 4/5 | 80 | 2/5 | 40 |
|  | Atropy | 1/10 | 10 | 0/5 | 0 | 0/5 | 0 | 0/5 | 0 | 0/5 | 0 |
|  | Degranulation | 1/10 | 10 | 0/5 | 0 | 1/5 | 20 | 1/5 | 20 | 3/5 | 60 |
| Liver | NSL | 2/10 | 20 | 1/5 | 20 | 0/5 | 0 | 0/5 | 0 | 0/5 | 0 |
|  | Inflammatory cell infiltration | 8/10 | 80 | 4/5 | 80 | 5/5 | 100 | 5/5 | 100 | 5/5 | 100 |
|  | Lipid droplets, Diffuse | 5/10 | 50 | 1/5 | 20 | 2/5 | 40 | 4/5 | 80 | 4/5 | 80 |
|  | Focal, Necrosis | 0/10 | 0 | 0/5 | 0 | 0/5 | 0 | 0/5 | 0 | 1/5 | 20 |
| Kidneys | NSL | 3/10 | 30 | 1/5 | 20 | 0/5 | 0 | 2/5 | 40 | 0/5 | 0 |
|  | Inflammatory cell infiltration | 7/10 | 70 | 4/5 | 80 | 5/5 | 100 | 3/5 | 60 | 5/5 | 100 |
|  | Regeneration, Tubules | 0/10 | 0 | 0/5 | 0 | 0/5 | 0 | 1/5 | 20 | 1/5 | 20 |
|  | Glomerulous Atropy | 0/10 | 0 | 0/5 | 0 | 1/5 | 20 | 0/5 | 0 | 0/5 | 0 |
| Testes | NSL | 10/10 | 100 | 5/5 | 100 | 5/5 | 100 | 5/5 | 100 | 5/5 | 100 |
| Epididymis | NSL | 1/10 | 10 | 0/5 | 0 | 0/5 | 0 | 3/5 | 60 | 2/5 | 40 |
|  | Inflammatory cell infiltration | 9/10 | 90 | 5/5 | 100 | 5/5 | 100 | 2/5 | 40 | 3/5 | 60 |

Supplement 4. Histopathological findings for female rats after 28-day oral administration of silver nanoparticles. NSL: No significant lesions found

| GROUP:  DOSE (mg/kg/day): | | Control  0 | | 10 nm  100 | | 10 nm  500 | | 25 nm  100 | | 25 nm  500 | |
| --- | --- | --- | --- | --- | --- | --- | --- | --- | --- | --- | --- |
| Number of Animals | | 10 | | 5 | | 5 | | 5 | | 5 | |
|  |  | N | % | N | % | N | % | N | % | N | % |
| Brain (Cerebrum, Cerebellum) | NSL | 10/10 | 100 | 5/5 | 100 | 5/5 | 100 | 5/5 | 100 | 5/5 | 100 |
| Lungs (Trachea) | NSL | 9/10 | 90 | 5/5 | 100 | 5/5 | 100 | 3/5 | 60 | 3/5 | 60 |
|  | Inflammatory cell infiltration | 1/10 | 10 | 0/5 | 0 | 0/5 | 0 | 2/5 | 40 | 2/5 | 40 |
| Spleen | NSL | 8/10 | 80 | 4/5 | 80 | 4/5 | 80 | 3/5 | 60 | 3/5 | 60 |
|  | Hypertropy | 1/10 | 10 | 0/5 | 0 | 0/5 | 0 | 0/5 | 0 | 0/5 | 0 |
|  | Degranulation | 1/10 | 10 | 0/5 | 0 | 1/5 | 20 | 2/5 | 40 | 2/5 | 40 |
|  | Atropy | 0/10 | 0 | 1/5 | 20 | 0/5 | 0 | 0/5 | 0 | 0/5 | 0 |
| Liver | NSL | 0/10 | 0 | 1/5 | 20 | 1/5 | 20 | 0/5 | 0 | 0/5 | 0 |
|  | Lipid droplets, Diffuse | 7/10 | 70 | 2/5 | 40 | 2/5 | 40 | 2/5 | 40 | 4/5 | 80 |
|  | Inflammatory cell infiltration | 8/10 | 80 | 4/5 | 80 | 3/5 | 60 | 4/5 | 80 | 5/5 | 100 |
|  | Lipid droplets,  Necrosis | 1/10 | 10 | 1/5 | 20 | 0/5 | 0 | 0/5 | 0 | 0/5 | 0 |
|  | Focal, Necrosis | 0/10 | 0 | 1/5 | 20 | 0/5 | 0 | 2/5 | 40 | 0/5 | 0 |
| Kidneys | NSL | 5/10 | 50 | 1/5 | 20 | 0/5 | 0 | 1/5 | 0 | 1/5 | 20 |
|  | Inflammatory cell infiltration | 4/10 | 40 | 3/5 | 60 | 3/5 | 60 | 4/5 | 80 | 4/5 | 80 |
|  | Hypertropy | 1/10 | 10 | 0/5 | 0 | 0/5 | 0 | 0/5 | 0 | 0/5 | 0 |
|  | Mineralization | 0/10 | 0 | 2/5 | 40 | 0/5 | 0 | 0/5 | 0 | 0/5 | 0 |
|  | Focal, Necrosis | 0/10 | 0 | 1/5 | 20 | 0/5 | 0 | 0/5 | 0 | 0/5 | 0 |
|  | Tubule Atropy | 0/10 | 0 | 0/5 | 0 | 5/5 | 100 | 0/5 | 0 | 0/5 | 0 |
| Ovaries | NSL | 10/10 | 100 | 5/5 | 100 | 4/5 | 80 | 5/5 | 100 | 5/5 | 100 |
|  | Parenchyma  Lipid droplets | 0/10 | 0 | 0/5 | 0 | 1/5 | 20 | 0/5 | 0 | 0/5 | 0 |
| Uterus | NSL | 10/10 | 100 | 5/5 | 100 | 5/5 | 100 | 5/5 | 100 | 5/5 | 100 |

Supplement 5. Histopathological findings for female rats after 1 month of recovery following 28-day oral administration of silver nanoparticles. NSL: No significant lesions found

| GROUP:  DOSE (mg/kg/day): | | Control  0 | | 10 nm  100 | | 10 nm  500 | | 25 nm  100 | | 25 nm  500 | |
| --- | --- | --- | --- | --- | --- | --- | --- | --- | --- | --- | --- |
| Number of Animals | | 10 | | 5 | | 5 | | 5 | | 5 | |
|  |  | N | % | N | % | N | % | N | % | N | % |
| Brain (Cerebrum, Cerebellum) | NSL | 9/10 | 90 | 5/5 | 100 | 5/5 | 100 | 5/5 | 100 | 5/5 | 100 |
|  | Cystic change | 1/10 | 10 | 0/5 | 0 | 0/5 | 0 | 0/5 | 0 | 0/5 | 0 |
| Lungs (Trachea) | NSL | 10/10 | 100 | 4/5 | 80 | 4/5 | 80 | 4/5 | 80 | 4/5 | 80 |
|  | Inflammatory cell infiltration | 0/10 | 0 | 1/10 | 10 | 1/5 | 20 | 1/5 | 20 | 1/5 | 20 |
| Spleen | NSL | 4/10 | 40 | 2/5 | 40 | 3/5 | 60 | 4/5 | 80 | 2/5 | 40 |
|  | Atropy | 4/10 | 40 | 1/5 | 20 | 2/5 | 40 | 0/5 | 0 | 2/5 | 40 |
|  | Degranulation | 2/10 | 20 | 3/5 | 60 | 0/5 | 0 | 1/5 | 20 | 1/5 | 20 |
| Liver | NSL | 2/10 | 20 | 0/5 | 0 | 1/5 | 20 | 0/5 | 0 | 0/5 | 0 |
|  | Lipid droplets, Diffuse | 4/10 | 40 | 1/5 | 20 | 2/5 | 40 | 2/5 | 40 | 2/5 | 40 |
|  | Inflammatory cell infiltration | 8/10 | 80 | 5/5 | 100 | 4/5 | 80 | 5/5 | 100 | 5/5 | 100 |
|  | Focal, Necrosis | 1/10 | 10 | 0/5 | 0 | 0/5 | 0 | 0/5 | 0 | 0/5 | 0 |
| Kidneys | NSL | 4/10 | 40 | 4/5 | 80 | 1/5 | 20 | 2/5 | 40 | 3/5 | 60 |
|  | Inflammatory cell infiltration | 5/10 | 50 | 1/5 | 20 | 3/5 | 60 | 3/5 | 60 | 2/5 | 40 |
|  | Focal, Necrosis | 1/10 | 10 | 0/5 | 0 | 1/5 | 20 | 0/5 | 0 | 0/5 | 0 |
|  | Tubule Degeneration | 0/10 | 0 | 0/5 | 0 | 0/5 | 0 | 1/5 | 20 | 0/5 | 0 |
| Ovaries | NSL | 10/10 | 100 | 5/5 | 100 | 5/5 | 100 | 5/5 | 100 | 5/5 | 100 |
| Uterus | NSL | 10/10 | 100 | 5/5 | 100 | 5/5 | 100 | 5/5 | 100 | 5/5 | 100 |

Supplement 6. Histopathological findings for female rats after 2 months of recovery following 28-day oral administration of silver nanoparticles. NSL: No significant lesions found

| GROUP:  DOSE (mg/kg/day): | | Control  0 | | 10 nm  100 | | 10 nm  500 | | 25 nm  100 | | 25 nm  500 | |
| --- | --- | --- | --- | --- | --- | --- | --- | --- | --- | --- | --- |
| Number of Animals | | 10 | | 5 | | 5 | | 5 | | 5 | |
|  |  | N | % | N | % | N | % | N | % | N | % |
| Brain (Cerebrum, Cerebellum) | NSL | 9/10 | 90 | 5/5 | 100 | 5/5 | 100 | 5/5 | 100 | 5/5 | 100 |
|  | Epithelium Degeneration | 1/10 | 10 | 0/5 | 0 | 0/5 | 0 | 0/5 | 0 | 0/5 | 0 |
| Lungs (Trachea) | NSL | 5/10 | 50 | 5/5 | 100 | 3/5 | 60 | 3/5 | 60 | 2/5 | 40 |
|  | Inflammatory cell infiltration | 5/10 | 50 | 0/5 | 0 | 2/5 | 40 | 2/5 | 40 | 3/5 | 60 |
|  | Hypertropy Bronchiole | 0/10 | 0 | 0/5 | 0 | 0/5 | 0 | 1/5 | 20 | 0/5 | 0 |
| Spleen | NSL | 9/10 | 90 | 4/5 | 80 | 4/5 | 80 | 3/5 | 60 | 4/5 | 80 |
|  | Degranulation | 1/10 | 10 | 1/5 | 20 | 1/5 | 20 | 2/5 | 40 | 1/5 | 20 |
| Liver | NSL | 1/10 | 10 | 1/5 | 20 | 0/5 | 0 | 1/5 | 20 | 1/5 | 20 |
|  | Lipid droplets, Diffuse | 6/10 | 60 | 2/5 | 40 | 1/5 | 20 | 1/5 | 20 | 3/5 | 60 |
|  | Inflammatory cell infiltration | 8/10 | 80 | 4/5 | 80 | 5/5 | 100 | 4/5 | 80 | 2/5 | 40 |
|  | Focal, Necrosis | 0/10 | 0 | 1/5 | 20 | 0/5 | 0 | 0/5 | 0 | 1/5 | 20 |
| Kidneys | NSL | 4/10 | 40 | 2/5 | 40 | 1/5 | 20 | 4/5 | 80 | 3/5 | 60 |
|  | Inflammatory cell infiltration | 5/10 | 50 | 2/5 | 40 | 3/5 | 60 | 0/5 | 0 | 2/5 | 40 |
|  | Mineralization | 4/10 | 40 | 2/5 | 40 | 2/5 | 40 | 1/5 | 20 | 0/5 | 0 |
| Ovaries | NSL | 9/10 | 90 | 5/5 | 100 | 5/5 | 100 | 5/5 | 100 | 5/5 | 100 |
|  | Benign Granulosa cell tumor | 1/10 | 10 | 0/5 | 0 | 0/5 | 0 | 0/5 | 0 | 0/5 | 0 |
| Uterus | NSL | 10/10 | 100 | 5/5 | 100 | 5/5 | 100 | 5/5 | 100 | 5/5 | 100 |

NSL: No significant lesions found

Supplement 7. Histological findings in liver and kidneys.

|  |  |
| --- | --- |
| A. Lipid droplets and inflammatory cell infiltration in liver in control after one month of recovery (200x) | B. Tubular regeneration in kidneys in control after one month of recovery (200x) |
| 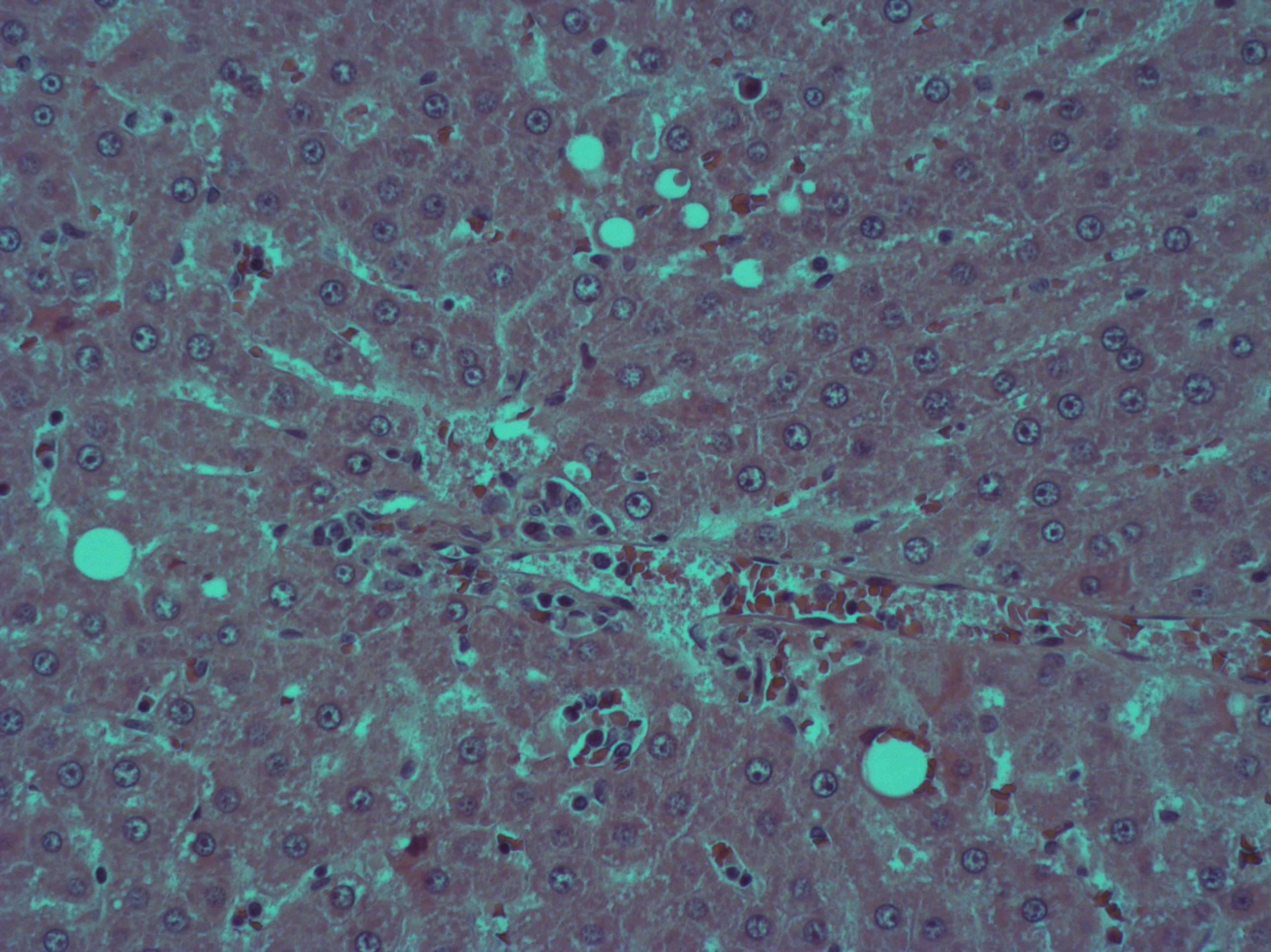 | 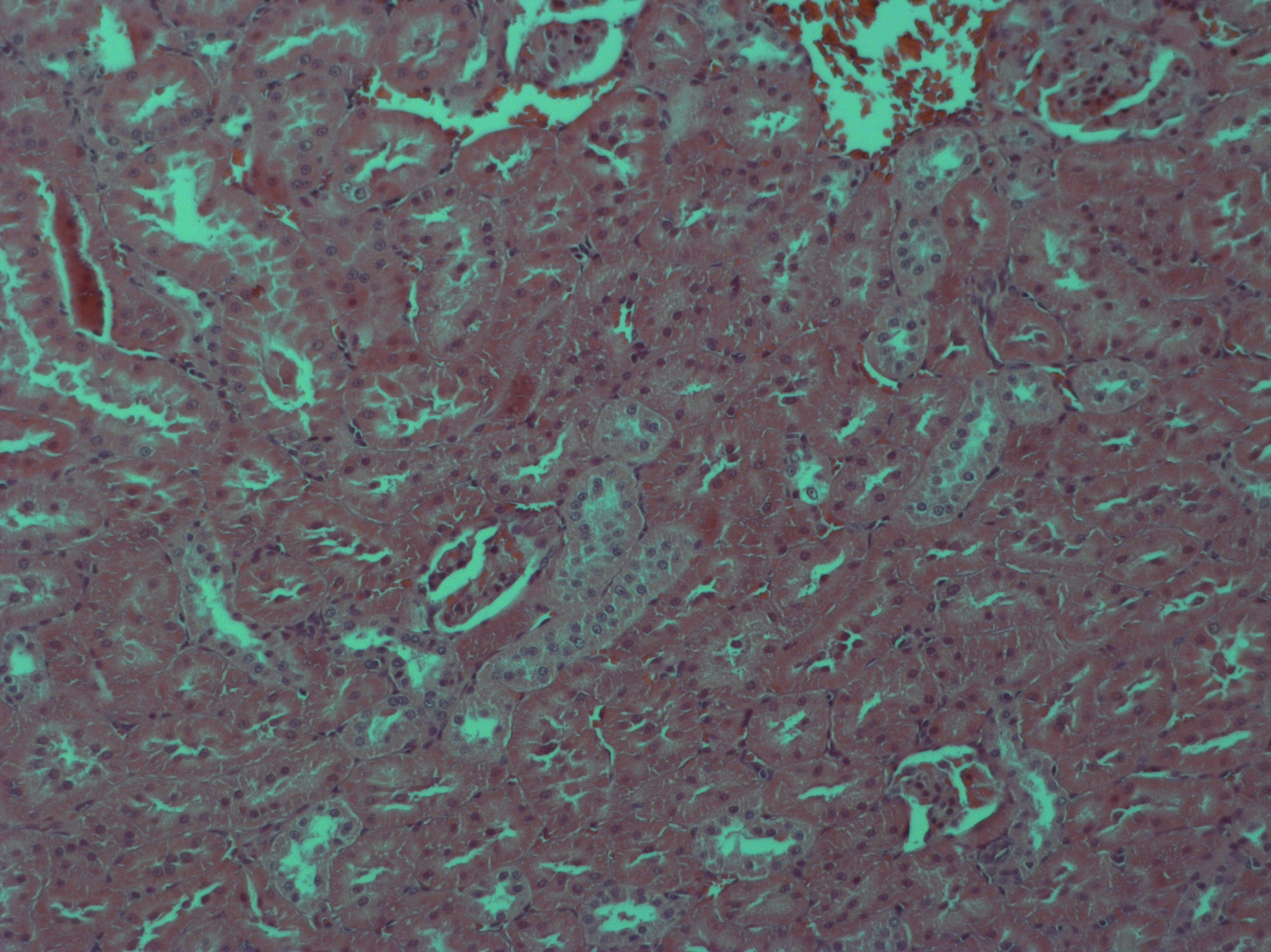 |

Supplement 8. Hematological values for male rats after 28-day oral administration of silver nanoparticles

|  | | | | | | | | | |
| --- | --- | --- | --- | --- | --- | --- | --- | --- | --- |
| TESTS | WBC^1^ | NE^2^ | | LY^3^ | | MO^4^ | | EO^5^ | |
| UNITS | K/μL | K/μL | | K/μL | | K/μL | | K/μL | |
| GROUP: Control (0㎎/㎏) | | |  | |  | |  | | |
| MEAN | 13.78 | 3.63 | | 9.47 | | 0.63 | | 0.04 | |
| S.D. | 2.70 | 0.73 | | 2.31 | | 0.16 | | 0.02 | |
| N | 9 | 9 | | 9 | | 9 | | 9 | |
| GROUP: 10 nm (100㎎/㎏) | | |  | |  | |  | |  |
| MEAN | 10.78 | 2.59 | | 7.61 | | 0.53 | | 0.05 | |
| S.D. | 2.66 | 1.30 | | 1.51 | | 0.22 | | 0.03 | |
| N | 5 | 5 | | 5 | | 5 | | 5 | |
| GROUP: 10 nm (500㎎/㎏) | | |  | |  | |  | |  |
| MEAN | 13.96 | 3.33 | | 9.97 | | 0.57 | | 0.07 | |
| S.D. | 5.29 | 1.30 | | 3.70 | | 0.27 | | 0.05 | |
| N# | 3 | 3 | | 3 | | 3 | | 3 | |
| GROUP 25 nm (100㎎/㎏) | | |  | |  | |  | |  |
| MEAN | 11.82 | 2.57 | | 8.63 | | 0.58 | | 0.03 | |
| S.D. | 4.77 | 0.86 | | 3.84 | | 0.11 | | 0.02 | |
| N | 5 | 5 | | 5 | | 5 | | 5 | |
| GROUP: 25 nm (500㎎/㎏) | | |  | |  | |  | |  |
| MEAN | 13.47 | 3.73 | | 9.09 | | 0.62 | | 0.03 | |
| S.D. | 3.73 | 1.10 | | 3.10 | | 0.18 | | 0.02 | |
| N# | 4 | 4 | | 4 | | 4 | | 4 | |

(Continued)

| TESTS | BA^6^ | NE^7^ | | LY^8^ | | MO^9^ | | EO^10^ | |
| --- | --- | --- | --- | --- | --- | --- | --- | --- | --- |
| UNITS | K/μL | % | | % | | % | | % | |
| GROUP: Control (0㎎/㎏) | | |  | |  | |  | | |
| MEAN | 0.01 | 26.78 | | 68.30 | | 4.62 | | 0.26 | |
| S.D. | 0.01 | 5.48 | | 5.48 | | 0.96 | | 0.13 | |
| N | 9 | 9 | | 9 | | 9 | | 9 | |
| GROUP: 10 nm (100㎎/㎏) | | |  | |  | |  | |  |
| MEAN | 0.01 | 23.04 | | 71.68 | | 4.80 | | 0.42 | |
| S.D. | 0.01 | 9.40 | | 10.28 | | 1.50 | | 0.23 | |
| N# | 5 | 5 | | 5 | | 5 | | 5 | |
| GROUP: 10 nm (500㎎/㎏) | | |  | |  | |  | |  |
| MEAN | 0.01 | 23.84 | | 71.61 | | 4.04 | | 0.45 | |
| S.D. | 0.01 | 1.15 | | 0.93 | | 0.75 | | 0.27 | |
|  | 3 | 3 | | 3 | | 3 | | 3 | |
| GROUP: 25 nm (100㎎/㎏) | | |  | |  | |  | |  |
| MEAN | 0.00 | 22.29 | | 72.21 | | 5.23 | | 0.23 | |
| S.D. | 0.01 | 3.26 | | 3.74 | | 1.09 | | 0.16 | |
| N | 5 | 5 | | 5 | | 5 | | 5 | |
| GROUP: 25 nm (500㎎/㎏) | | |  | |  | |  | |  |
| MEAN | 0.01 | 28.32 | | 66.73 | | 4.66 | | 0.26 | |
| S.D. | 0.01 | 6.43 | | 7.20 | | 0.86 | | 0.13 | |
| N# | 4 | 4 | | 4 | | 4 | | 4 | |

Continued,

|  |  | |  | |  | |  | |  | | |
| --- | --- | --- | --- | --- | --- | --- | --- | --- | --- | --- | --- |
| TESTS | | BA^11^ | | RBC^12^ | | Hb^14^ | | HCT^14^ | | MCV^15^ | |
| UNITS | | % | | M/μL | | g/dL | | % | | fL | |
| GROUP: CONTROL(0㎎/㎏) | | | | |  | |  | |  | | |
| MEAN | | 0.03 | | 7.25 | | 17.24 | | 32.50 | | 44.82 | |
| S.D. | | 0.04 | | 0.27 | | 0.96 | | 1.99 | | 2.13 | |
| N | | 9 | | 9 | | 9 | | 9 | | 9 | |
| GROUP: 10 nm(100㎎/㎏) | | | | |  | |  | |  | |  |
| MEAN | | 0.05 | | 6.38 | | 14.52 | | 28.32 | | 44.72 | |
| S.D. | | 0.04 | | 1.99 | | 5.70 | | 8.43 | | 1.33 | |
| N | | 5 | | 5 | | 5 | | 5 | | 5 | |
| GROUP: 10 nm (500㎎/㎏) | | | | |  | |  | |  | |  |
| MEAN | | 0.07 | | 7.39 | | 16.93 | | 33.03 | | 44.77 | |
| S.D. | | 0.06 | | 0.58 | | 1.24 | | 0.99 | | 2.32 | |
| N# | | 3 | | 3 | | 3 | | 3 | | 3 | |
| GROUP: 25 nm0 (100㎎/㎏) | | | | |  | |  | |  | |  |
| MEAN | | 0.04 | | 8.32 | | 17.10 | | 37.26 | | 44.84 | |
| S.D. | | 0.05 | | 1.09 | | 0.60 | | 5.04 | | 2.05 | |
| N | | 5 | | 5 | | 5 | | 5 | | 5 | |
| GROUP: 25 nm (500㎎/㎏) | | | | |  | |  | |  | |  |
| MEAN | | 0.04 | | 7.46 | | 16.48 | | 33.35 | | 44.73 | |
| S.D. | | 0.05 | | 0.29 | | 0.76 | | 1.15 | | 1.45 | |
| N# | | 4 | | 4 | | 4 | | 4 | | 4 | |

Continued

|  |  | |  | |  | |  | |  | | |
| --- | --- | --- | --- | --- | --- | --- | --- | --- | --- | --- | --- |
| TESTS | | MCH^16^ | | MCHC^17^ | | RDW^18^ | | PLT^19^ | | MPV^20^ | |
| UNITS | | pg | | g/dL | | % | | K/μL | | fL | |
| GROUP: CONTROL(0㎎/㎏) | | | | |  | |  | |  | | |
| MEAN | | 23.80 | | 53.28 | | 19.76 | | 785.11 | | 3.67 | |
| S.D. | | 1.15 | | 4.94 | | 1.42 | | 46.76 | | 0.21 | |
| N# | | 9 | | 9 | | 9 | | 9 | | 9 | |
| GROUP: 10 nm(100㎎/㎏) | | | | |  | |  | |  | |  |
| MEAN | | 22.00 | | 49.40 | | 19.90 | | 695.00 | | 3.58 | |
| S.D. | | 3.24 | | 8.40 | | 0.97 | | 128.89 | | 0.36 | |
| N | | 5 | | 5 | | 5 | | 5 | | 5 | |
| GROUP: 10 nm (500㎎/㎏) | | | | |  | |  | |  | |  |
| MEAN | | 22.90 | | 51.20 | | 18.37 | | 803.33 | | 3.17 | |
| S.D. | | 0.36 | | 2.27 | | 1.29 | | 310.54 | | 0.64 | |
| N# | | 3 | | 3 | | 3 | | 3 | | 3 | |
| GROUP: 25 nm (100㎎/㎏) | | | | |  | |  | |  | |  |
| MEAN | | 20.80 | | 46.48 | | 19.28 | | 880.00 | | 3.36 | |
| S.D. | | 2.67 | | 5.59 | | 2.32 | | 111.29 | | 0.40 | |
| N | | 5 | | 5 | | 5 | | 5 | | 5 | |
| GROUP: 25 nm (500㎎/㎏) | | | | |  | |  | |  | |  |
| MEAN | | 22.08 | | 49.40 | | 18.75 | | 823.75 | | 3.35 | |
| S.D. | | 0.57 | | 1.12 | | 0.33 | | 95.41 | | 0.26 | |
| N# | | 4 | | 4 | | 4 | | 4 | | 4 | |

1, White blood cells; 2, Neutrophils; 3, Lymphocytes; 4, Monocytes; 5, Eosinophils 6, Basophils; 7, Percent of neutrophils; 8, Percent of lymphocytes; 9, Percent of monocytes; 10, Percent of eosinophils, 11, Percent of basophils; 12, Red blood cells; 13, Hemoglobin; 14, Hematocrit; 15, Mean corpuscular volume, 16, Mean corpuscular hemoglobin; 17, Mean corpuscular hemoglobin concentration;

18, Red cell distribution width; 19, Platelets; 20, Mean platelet volume, S.D.: Standard Deviation, N: Number of animals; #, sample loss due to blood coagulation

Supplement 9. Hematological values for male rats after 1 month of recovery following 28-day oral administration of silver nanoparticles.

|  |  | |  | |  | |  | |  | | |
| --- | --- | --- | --- | --- | --- | --- | --- | --- | --- | --- | --- |
| TESTS | | WBC^1^ | | NE^2^ | | LY^3^ | | MO^4^ | | EO^5^ | |
| UNITS | | K/μL | | K/μL | | K/μL | | K/μL | | K/μL | |
| Control (0㎎/㎏) | | | | |  | |  | |  | | |
| MEAN | | 14.02 | | 4.04 | | 9.22 | | 0.71 | | 0.05 | |
| S.D. | | 4.40 | | 1.71 | | 3.15 | | 0.34 | | 0.05 | |
| N | | 10 | | 10 | | 10 | | 10 | | 10 | |
| 10 nm (100㎎/㎏) | | | | |  | |  | |  | |  |
| MEAN | | 15.46 | | 4.33 | | 10.26 | | 0.81 | | 0.05 | |
| S.D. | | 4.76 | | 1.04 | | 3.80 | | 0.34 | | 0.03 | |
| N | | 5 | | 5 | | 5 | | 5 | | 5 | |
| 10 nm (500㎎/㎏) | | | | |  | |  | |  | |  |
| MEAN | | 14.56 | | 4.18 | | 9.58 | | 0.76 | | 0.04 | |
| S.D. | | 6.24 | | 2.01 | | 3.98 | | 0.30 | | 0.04 | |
| N | | 5 | | 5 | | 5 | | 5 | | 5 | |
| 25 nm (100㎎/㎏) | | | | |  | |  | |  | |  |
| MEAN | | 13.39 | | 4.77 | | 7.75 | | 0.81 | | 0.04 | |
| S.D. | | 4.34 | | 1.91 | | 2.44 | | 0.26 | | 0.04 | |
| N | | 5 | | 5 | | 5 | | 5 | | 5 | |
| 25 nm (500㎎/㎏) | | | | |  | |  | |  | |  |
| MEAN | | 12.89 | | 3.78 | | 8.20 | | 0.85 | | 0.06 | |
| S.D. | | 2.53 | | 0.57 | | 2.18 | | 0.33 | | 0.04 | |
| N | | 5 | | 5 | | 5 | | 5 | | 5 | |

Continued

|  |  | |  | |  | |  | |  | | |
| --- | --- | --- | --- | --- | --- | --- | --- | --- | --- | --- | --- |
| TESTS | | BA^6^ | | NE^7^ | | LY^8^ | | MO^9^ | | EO^10^ | |
| UNITS | | K/μL | | % | | % | | % | | % | |
| GROUP: G1-1-1 / G1-2-1 (0㎎/㎏) | | | | |  | |  | |  | | |
| MEAN | | 0.01 | | 28.70 | | 65.89 | | 5.05 | | 0.32 | |
| S.D. | | 0.01 | | 8.83 | | 9.55 | | 1.66 | | 0.30 | |
| N | | 10 | | 10 | | 10 | | 10 | | 10 | |
| GROUP: G2-1-1 (100㎎/㎏) | | | | |  | |  | |  | |  |
| MEAN | | 0.00 | | 28.58 | | 65.75 | | 5.31 | | 0.32 | |
| S.D. | | 0.01 | | 3.92 | | 5.57 | | 2.12 | | 0.19 | |
| N | | 5 | | 5 | | 5 | | 5 | | 5 | |
| GROUP: G3-1-1 (500㎎/㎏) | | | | |  | |  | |  | |  |
| MEAN | | 0.00 | | 27.82 | | 66.66 | | 5.27 | | 0.24 | |
| S.D. | | 0.00 | | 3.71 | | 4.02 | | 0.54 | | 0.21 | |
| N | | 5 | | 5 | | 5 | | 5 | | 5 | |
| GROUP: G2-2-1 (100㎎/㎏) | | | | |  | |  | |  | |  |
| MEAN | | 0.00 | | 35.37 | | 58.14 | | 6.12 | | 0.33 | |
| S.D. | | 0.01 | | 5.20 | | 4.62 | | 0.90 | | 0.21 | |
| N | | 5 | | 5 | | 5 | | 5 | | 5 | |
| GROUP: G3-2-1 (500㎎/㎏) | | | | |  | |  | |  | |  |
| MEAN | | 0.00 | | 30.05 | | 62.88 | | 6.58 | | 0.45 | |
| S.D. | | 0.01 | | 6.60 | | 6.56 | | 1.79 | | 0.32 | |
| N | | 5 | | 5 | | 5 | | 5 | | 5 | |

Continued

|  |  | |  | |  | |  | |  | | |
| --- | --- | --- | --- | --- | --- | --- | --- | --- | --- | --- | --- |
| TESTS | | BA^11^ | | RBC^12^ | | Hb^14^ | | HCT^14^ | | MCV^15^ | |
| UNITS | | % | | M/μL | | g/dL | | % | | fL | |
| GROUP: Control (0㎎/㎏) | | | | |  | |  | |  | | |
| MEAN | | 0.05 | | 7.50 | | 18.85 | | 35.42 | | 47.20 | |
| S.D. | | 0.05 | | 0.30 | | 0.95 | | 1.93 | | 1.64 | |
| N | | 10 | | 10 | | 10 | | 10 | | 10 | |
| GROUP: 10 nm (100㎎/㎏) | | | | |  | |  | |  | |  |
| MEAN | | 0.04 | | 7.14 | | 18.42 | | 35.22 | | 49.32 | |
| S.D. | | 0.03 | | 0.53 | | 1.56 | | 2.64 | | 1.73 | |
| N | | 5 | | 5 | | 5 | | 5 | | 5 | |
| GROUP: 10 nm (500㎎/㎏) | | | | |  | |  | |  | |  |
| MEAN | | 0.01 | | 7.58 | | 19.20 | | 35.90 | | 47.38 | |
| S.D. | | 0.01 | | 0.20 | | 0.76 | | 1.42 | | 1.60 | |
| N | | 5 | | 5 | | 5 | | 5 | | 5 | |
| GROUP: 25 nm (100㎎/㎏) | | | | |  | |  | |  | |  |
| MEAN | | 0.04 | | 7.45 | | 19.32 | | 36.00 | | 48.38 | |
| S.D. | | 0.04 | | 0.17 | | 0.18 | | 0.74 | | 1.88 | |
| N | | 5 | | 5 | | 5 | | 5 | | 5 | |
| GROUP: 25 nm (500㎎/㎏) | | | | |  | |  | |  | |  |
| MEAN | | 0.04 | | 7.53 | | 18.82 | | 36.76 | | 48.82 | |
| S.D. | | 0.06 | | 0.32 | | 0.93 | | 1.37 | | 1.28 | |
| N | | 5 | | 5 | | 5 | | 5 | | 5 | |

Continued

|  |  | |  | |  | |  | |  | | |
| --- | --- | --- | --- | --- | --- | --- | --- | --- | --- | --- | --- |
| TESTS | | MCH^16^ | | MCHC^17^ | | RDW^18^ | | PLT^19^ | | MPV^20^ | |
| UNITS | | pg | | g/dL | | % | | K/μL | | fL | |
| GROUP: Control (0㎎/㎏) | | | | |  | |  | |  | | |
| MEAN | | 25.13 | | 53.25 | | 17.39 | | 785.40 | | 4.08 | |
| S.D. | | 0.71 | | 1.82 | | 0.46 | | 80.40 | | 0.41 | |
| N | | 10 | | 10 | | 10 | | 10 | | 10 | |
| GROUP: 10 nm (100㎎/㎏) | | | | |  | |  | |  | |  |
| MEAN | | 25.80 | | 52.30 | | 16.92 | | 716.40 | | 4.08 | |
| S.D. | | 0.95 | | 2.14 | | 0.71 | | 70.36 | | 0.54 | |
| N | | 5 | | 5 | | 5 | | 5 | | 5 | |
| GROUP: 10 nm (500㎎/㎏) | | | | |  | |  | |  | |  |
| MEAN | | 25.32 | | 53.52 | | 17.90 | | 818.40 | | 3.98 | |
| S.D. | | 0.66 | | 0.64 | | 0.28 | | 102.60 | | 0.16 | |
| N | | 5 | | 5 | | 5 | | 5 | | 5 | |
| GROUP: 25 nm (100㎎/㎏) | | | | |  | |  | |  | |  |
| MEAN | | 25.96 | | 53.66 | | 16.88 | | 720.20 | | 4.44 | |
| S.D. | | 0.60 | | 1.34 | | 0.89 | | 87.74 | | 0.61 | |
| N | | 5 | | 5 | | 5 | | 5 | | 5 | |
| GROUP: 25 nm (500㎎/㎏) | | | | |  | |  | |  | |  |
| MEAN | | 25.00 | | 51.16 | | 17.36 | | 808.00 | | 3.84 | |
| S.D. | | 0.56 | | 1.08 | | 0.46 | | 71.04 | | 0.35 | |
| N | | 5 | | 5 | | 5 | | 5 | | 5 | |

1, White blood cells; 2, Neutrophils; 3, Lymphocytes; 4, Monocytes; 5, Eosinophils, 6, Basophils; 7, Percent of neutrophils; 8, Percent of lymphocytes; 9, Percent of monocytes; 10, Percent of eosinophils, 11, Percent of basophils; 12, Red blood cells; 13, Hemoglobin; 14, Hematocrit; 15, Mean corpuscular volume, 16, Mean corpuscular hemoglobin; 17, Mean corpuscular hemoglobin concentration;

18, Red cell distribution width; 19, Platelets; 20, Mean platelet volume, S.D.: Standard Deviation, N: Number of animals

Supplement 10. Hematological values for male rats after 2 months of recovery following 28-day oral administration of silver nanoparticles

|  |  | |  | |  | |  | |  | | |
| --- | --- | --- | --- | --- | --- | --- | --- | --- | --- | --- | --- |
| TESTS | | WBC^1^ | | NE^2^ | | LY^3^ | | MO^4^ | | EO^5^ | |
| UNITS | | K/μL | | K/μL | | K/μL | | K/μL | | K/μL | |
| GROUP: Control (0㎎/㎏) | | | | |  | |  | |  | | |
| MEAN | | 14.65 | | 4.26 | | 9.56 | | 0.57 | | 0.17 | |
| S.D. | | 3.65 | | 1.14 | | 3.09 | | 0.11 | | 0.11 | |
| N | | 10 | | 10 | | 10 | | 10 | | 10 | |
| GROUP: 10 nm (100㎎/㎏) | | | | |  | |  | |  | |  |
| MEAN | | 15.45 | | 4.82 | | 9.84 | | 0.63 | | 0.12 | |
| S.D. | | 2.45 | | 0.86 | | 2.13 | | 0.14 | | 0.08 | |
| N | | 5 | | 5 | | 5 | | 5 | | 5 | |
| GROUP: 10 nm (500㎎/㎏) | | | | |  | |  | |  | |  |
| MEAN | | 14.79 | | 4.57 | | 9.57 | | 0.53 | | 0.11 | |
| S.D. | | 3.73 | | 1.66 | | 2.22 | | 0.20 | | 0.05 | |
| N | | 5 | | 5 | | 5 | | 5 | | 5 | |
| GROUP: 25 nm (100㎎/㎏) | | | | |  | |  | |  | |  |
| MEAN | | 12.35 | | 4.05 | | 7.54 | | 0.54 | | 0.16 | |
| S.D. | | 4.33 | | 1.93 | | 2.06 | | 0.24 | | 0.20 | |
| N | | 5 | | 5 | | 5 | | 5 | | 5 | |
| GROUP: 25 nm (500㎎/㎏) | | | | |  | |  | |  | |  |
| MEAN | | 11.56 | | 3.56 | | 7.53 | | 0.39 | | 0.07 | |
| S.D. | | 2.74 | | 1.55 | | 1.44 | | 0.09 | | 0.04 | |
| N | | 5 | | 5 | | 5 | | 5 | | 5 | |
| Continued | | | | | | | | | | | |
| TESTS | | BA^6^ | | NE^7^ | | LY^8^ | | MO^9^ | | EO^10^ | |
| UNITS | | K/μL | | % | | % | | % | | % | |
| GROUP: Control (0㎎/㎏) | | | | |  | |  | |  | | |
| MEAN | | 0.05 | | 29.73 | | 64.82 | | 3.97 | | 1.12 | |
| S.D. | | 0.05 | | 8.28 | | 7.95 | | 0.69 | | 0.65 | |
| N | | 10 | | 10 | | 10 | | 10 | | 10 | |
| GROUP: 10 nm (100㎎/㎏) | | | | |  | |  | |  | |  |
| MEAN | | 0.04 | | 31.49 | | 63.34 | | 4.16 | | 0.77 | |
| S.D. | | 0.04 | | 5.38 | | 6.38 | | 1.15 | | 0.45 | |
| N | | 5 | | 5 | | 5 | | 5 | | 5 | |
| GROUP: 10 nm (500㎎/㎏) | | | | |  | |  | |  | |  |
| MEAN | | 0.01 | | 30.50 | | 65.17 | | 3.52 | | 0.72 | |
| S.D. | | 0.01 | | 5.36 | | 5.44 | | 0.69 | | 0.30 | |
| N | | 5 | | 5 | | 5 | | 5 | | 5 | |
| GROUP: 25 nm (100㎎/㎏) | | | | |  | |  | |  | |  |
| MEAN | | 0.06 | | 31.61 | | 62.66 | | 4.29 | | 1.06 | |
| S.D. | | 0.11 | | 7.87 | | 8.12 | | 0.89 | | 0.94 | |
| N | | 5 | | 5 | | 5 | | 5 | | 5 | |
| GROUP: 25 nm (500㎎/㎏) | | | | |  | |  | |  | |  |
| MEAN | | 0.01 | | 29.66 | | 66.19 | | 3.44 | | 0.59 | |
| S.D. | | 0.01 | | 7.48 | | 7.44 | | 0.70 | | 0.23 | |
| N | | 5 | | 5 | | 5 | | 5 | | 5 | |

Continued

|  |  | |  | |  | |  | |  | | |
| --- | --- | --- | --- | --- | --- | --- | --- | --- | --- | --- | --- |
| TESTS | | BA^11^ | | RBC^12^ | | Hb^14^ | | HCT^14^ | | MCV^15^ | |
| UNITS | | % | | M/μL | | g/dL | | % | | fL | |
| GROUP: Control (0㎎/㎏) | | | | |  | |  | |  | | |
| MEAN | | 0.37 | | 7.77 | | 15.86 | | 36.42 | | 47.07 | |
| S.D. | | 0.32 | | 0.50 | | 0.45 | | 1.03 | | 3.18 | |
| N | | 10 | | 10 | | 10 | | 10 | | 10 | |
| GROUP: 10 nm (100㎎/㎏) | | | | |  | |  | |  | |  |
| MEAN | | 0.25 | | 8.24 | | 16.48 | | 37.60 | | 45.64 | |
| S.D. | | 0.24 | | 0.52 | | 0.96 | | 1.71 | | 1.30 | |
| N | | 5 | | 5 | | 5 | | 5 | | 5 | |
| GROUP: 10 nm (500㎎/㎏) | | | | |  | |  | |  | |  |
| MEAN | | 0.09 | | 7.62 | | 15.70 | | 35.96 | | 47.56 | |
| S.D. | | 0.10 | | 0.78 | | 0.80 | | 2.56 | | 5.70 | |
| N | | 5 | | 5 | | 5 | | 5 | | 5 | |
| GROUP: 25 nm (100㎎/㎏) | | | | |  | |  | |  | |  |
| MEAN | | 0.38 | | 8.05 | | 15.48 | | 36.34 | | 45.16 | |
| S.D. | | 0.54 | | 0.26 | | 0.68 | | 2.03 | | 2.65 | |
| N | | 5 | | 5 | | 5 | | 5 | | 5 | |
| GROUP: 25 nm (500㎎/㎏) | | | | |  | |  | |  | |  |
| MEAN | | 0.11 | | 8.32 | | 15.74 | | 37.38 | | 44.94 | |
| S.D. | | 0.07 | | 0.28 | | 0.85 | | 1.58 | | 1.39 | |
| N# | | 4 | | 4 | | 4 | | 4 | | 4 | |
| Continued | | | | | | | | | | | |
|  |  | |  | |  | |  | |  | | |
| TESTS | | MCH^16^ | | MCHC^17^ | | RDW^18^ | | PLT^19^ | | MPV^20^ | |
| UNITS | | pg | | g/dL | | % | | K/μL | | fL | |
| GROUP: Control (0㎎/㎏) | | | | |  | |  | |  | | |
| MEAN | | 20.48 | | 43.56 | | 17.09 | | 864.60 | | 6.73 | |
| S.D. | | 1.29 | | 1.25 | | 2.27 | | 183.17 | | 0.85 | |
| N | | 10 | | 10 | | 10 | | 10 | | 10 | |
| GROUP: 10 nm (100㎎/㎏) | | | | |  | |  | |  | |  |
| MEAN | | 20.00 | | 43.82 | | 16.88 | | 1008.20 | | 6.60 | |
| S.D. | | 0.71 | | 1.10 | | 0.49 | | 193.02 | | 0.86 | |
| N | | 5 | | 5 | | 5 | | 5 | | 5 | |
| GROUP: 10 nm (500㎎/㎏) | | | | |  | |  | |  | |  |
| MEAN | | 20.72 | | 43.76 | | 17.86 | | 901.00 | | 6.68 | |
| S.D. | | 1.72 | | 1.69 | | 2.85 | | 212.30 | | 0.76 | |
| N | | 5 | | 5 | | 5 | | 5 | | 5 | |
| GROUP: 25 nm (100㎎/㎏) | | | | |  | |  | |  | |  |
| MEAN | | 19.24 | | 42.62 | | 17.18 | | 1055.40 | | 7.24 | |
| S.D. | | 0.83 | | 1.36 | | 1.12 | | 150.75 | | 0.13 | |
| N | | 5 | | 5 | | 5 | | 5 | | 5 | |
| GROUP 25 nm (500㎎/㎏) | | | | |  | |  | |  | |  |
| MEAN | | 18.92 | | 42.12 | | 17.10 | | 1127.20 | | 7.16 | |
| S.D. | | 0.68 | | 1.34 | | 0.90 | | 89.04 | | 0.33 | |
| N | | 4 | | 4 | | 4 | | 4 | | 4 | |

1, White blood cells; 2, Neutrophils; 3, Lymphocytes; 4, Monocytes; 5, Eosinophils, 6, Basophils; 7, Percent of neutrophils; 8, Percent of lymphocytes; 9, Percent of monocytes; 10, Percent of eosinophils, 11, Percent of basophils; 12, Red blood cells; 13, Hemoglobin; 14, Hematocrit; 15, Mean corpuscular volume, 16, Mean corpuscular hemoglobin; 17, Mean corpuscular hemoglobin concentration; S.D.: Standard Deviation, N: Number of animals, ; #, sample loss due to blood coagulation

Supplement 11. Hematological values for female rats after 28-day oral administration of silver nanoparticles

|  |  | |  | |  | |  | |  | | |
| --- | --- | --- | --- | --- | --- | --- | --- | --- | --- | --- | --- |
| TESTS | | WBC^1^ | | NE^2^ | | LY^3^ | | MO^4^ | | EO^5^ | |
| UNITS | | K/μL | | K/μL | | K/μL | | K/μL | | K/μL | |
| GROUP: CONTROL(0㎎/㎏) | | | | |  | |  | |  | | |
| MEAN | | 9.14 | | 2.01 | | 6.74 | | 0.36 | | 0.03 | |
| S.D. | | 2.04 | | 0.75 | | 1.56 | | 0.06 | | 0.03 | |
| N | | 10 | | 10 | | 10 | | 10 | | 10 | |
| GROUP: 10 nm (100㎎/㎏) | | | | |  | |  | |  | |  |
| MEAN | | 7.12 | | 1.59 | | 5.27 | | 0.25 | | 0.02 | |
| S.D. | | 1.30 | | 0.54 | | 0.97 | | 0.14 | | 0.01 | |
| N | | 5 | | 5 | | 5 | | 5 | | 5 | |
| GROUP: 10 nm (500㎎/㎏) | | | | |  | |  | |  | |  |
| MEAN | | 10.57 | | 2.36 | | 7.83 | | 0.37 | | 0.01 | |
| S.D. | | 2.17 | | 0.83 | | 1.57 | | 0.17 | | 0.01 | |
| N | | 5 | | 5 | | 5 | | 5 | | 5 | |
| GROUP: 25 nm (100㎎/㎏) | | | | |  | |  | |  | |  |
| MEAN | | 10.63 | | 2.23 | | 8.04 | | 0.33 | | 0.02 | |
| S.D. | | 5.47 | | 1.42 | | 3.97 | | 0.13 | | 0.01 | |
| N | | 5 | | 5 | | 5 | | 5 | | 5 | |
| GROUP: 25 nm (500㎎/㎏) | | | | |  | |  | |  | |  |
| MEAN | | 8.32 | | 1.71 | | 6.23 | | 0.33 | | 0.04 | |
| S.D. | | 1.85 | | 0.83 | | 1.84 | | 0.09 | | 0.02 | |
| N# | | 4 | | 4 | | 4 | | 4 | | 4 | |
| Continued | | | | | | | | | | | |
|  |  | |  | |  | |  | |  | | |
| TESTS | | BA^6^ | | NE^7^ | | LY^8^ | | MO^9^ | | EO^10^ | |
| UNITS | | K/μL | | % | | % | | % | | % | |
| GROUP: CONTROL(0㎎/㎏) | | | | |  | |  | |  | | |
| MEAN | | 0.01 | | 21.83 | | 73.73 | | 4.04 | | 0.32 | |
| S.D. | | 0.01 | | 5.82 | | 5.93 | | 0.92 | | 0.27 | |
| N | | 10 | | 10 | | 10 | | 10 | | 10 | |
| GROUP: 10 nm (100㎎/㎏) | | | | |  | |  | |  | |  |
| MEAN | | 0.00 | | 22.10 | | 74.38 | | 3.24 | | 0.21 | |
| S.D. | | 0.01 | | 6.77 | | 7.58 | | 1.52 | | 0.11 | |
| N | | 5 | | 5 | | 5 | | 5 | | 5 | |
| GROUP: 10 nm (500㎎/㎏) | | | | |  | |  | |  | |  |
| MEAN | | 0.00 | | 22.03 | | 74.33 | | 3.50 | | 0.12 | |
| S.D. | | 0.00 | | 5.53 | | 5.28 | | 1.30 | | 0.11 | |
| N | | 5 | | 5 | | 5 | | 5 | | 5 | |
| GROUP: 25 nm (100㎎/㎏) | | | | |  | |  | |  | |  |
| MEAN | | 0.01 | | 20.15 | | 76.25 | | 3.35 | | 0.19 | |
| S.D. | | 0.01 | | 2.81 | | 2.89 | | 1.11 | | 0.11 | |
| N | | 5 | | 5 | | 5 | | 5 | | 5 | |
| GROUP: 25 nm (500㎎/㎏) | | | | |  | |  | |  | |  |
| MEAN | | 0.01 | | 20.59 | | 74.74 | | 3.99 | | 0.51 | |
| S.D. | | 0.01 | | 9.45 | | 10.22 | | 0.94 | | 0.41 | |
| N# | | 4 | | 4 | | 4 | | 4 | | 4 | |

Continued

|  |  | |  | |  | |  | |  | | |
| --- | --- | --- | --- | --- | --- | --- | --- | --- | --- | --- | --- |
| TESTS | | BA^11^ | | RBC^12^ | | Hb^14^ | | HCT^14^ | | MCV^15^ | |
| UNITS | | % | | M/μL | | g/dL | | % | | fL | |
| GROUP: CONTROL(0㎎/㎏) | | | | |  | |  | |  | | |
| MEAN | | 0.08 | | 7.10 | | 17.07 | | 31.10 | | 43.81 | |
| S.D. | | 0.09 | | 0.30 | | 1.02 | | 1.34 | | 1.28 | |
| N | | 10 | | 10 | | 10 | | 10 | | 10 | |
| GROUP: 10 nm (100㎎/㎏) | | | | |  | |  | |  | |  |
| MEAN | | 0.07 | | 6.76 | | 16.64 | | 29.66 | | 43.90 | |
| S.D. | | 0.08 | | 0.13 | | 0.71 | | 1.14 | | 1.95 | |
| N | | 5 | | 5 | | 5 | | 5 | | 5 | |
| GROUP: 10 nm (500㎎/㎏) | | | | |  | |  | |  | |  |
| MEAN | | 0.02 | | 7.13 | | 18.02 | | 32.08 | | 45.04 | |
| S.D. | | 0.02 | | 0.56 | | 0.75 | | 1.89 | | 1.28 | |
| N | | 5 | | 5 | | 5 | | 5 | | 5 | |
| GROUP: 25 nm (100㎎/㎏) | | | | |  | |  | |  | |  |
| MEAN | | 0.06 | | 7.06 | | 17.20 | | 31.54 | | 44.70 | |
| S.D. | | 0.04 | | 0.35 | | 1.07 | | 1.38 | | 1.05 | |
| N | | 5 | | 5 | | 5 | | 5 | | 5 | |
| GROUP: 25 nm (500㎎/㎏) | | | | |  | |  | |  | |  |
| MEAN | | 0.17 | | 7.05 | | 16.68 | | 31.00 | | 44.05 | |
| S.D. | | 0.15 | | 0.89 | | 1.82 | | 3.85 | | 1.34 | |
| N# | | 4 | | 4 | | 4 | | 4 | | 4 | |
| Continued | | | | | | | | | | | |
|  |  | |  | |  | |  | |  | | |
| TESTS | | MCH^16^ | | MCHC^17^ | | RDW^18^ | | PLT^19^ | | MPV^20^ | |
| UNITS | | pg | | g/dL | | % | | K/μL | | fL | |
| GROUP: CONTROL(0㎎/㎏) | | | | |  | |  | |  | | |
| MEAN | | 24.02 | | 54.87 | | 15.25 | | 985.40 | | 4.00 | |
| S.D. | | 1.16 | | 2.33 | | 0.44 | | 281.95 | | 0.57 | |
| N | | 10 | | 10 | | 10 | | 10 | | 10 | |
| GROUP: 10 nm (100㎎/㎏) | | | | |  | |  | |  | |  |
| MEAN | | 24.62 | | 56.14 | | 14.94 | | 743.60 | | 4.54 | |
| S.D. | | 1.32 | | 1.92 | | 0.32 | | 148.30 | | 0.36 | |
| N | | 5 | | 5 | | 5 | | 5 | | 5 | |
| GROUP: 10 nm (500㎎/㎏) | | | | |  | |  | |  | |  |
| MEAN | | 25.32 | | 56.22 | | 15.40 | | 886.80 | | 4.38 | |
| S.D. | | 0.96 | | 1.31 | | 0.29 | | 231.13 | | 0.56 | |
| N | | 5 | | 5 | | 5 | | 5 | | 5 | |
| GROUP: 25 nm (100㎎/㎏) | | | | |  | |  | |  | |  |
| MEAN | | 24.36 | | 54.56 | | 15.24 | | 878.40 | | 4.42 | |
| S.D. | | 1.01 | | 2.53 | | 0.43 | | 237.93 | | 0.95 | |
| N | | 5 | | 5 | | 5 | | 5 | | 5 | |
| GROUP: 25 nm (500㎎/㎏) | | | | |  | |  | |  | |  |
| MEAN | | 23.73 | | 53.88 | | 15.23 | | 831.00 | | 3.93 | |
| S.D, | | 0.61 | | 1.82 | | 0.28 | | 549.19 | | 0.62 | |
| N# | | 4 | | 4 | | 4 | | 4 | | 4 | |

1, White blood cells; 2, Neutrophils; 3, Lymphocytes; 4, Monocytes; 5, Eosinophils, 6, Basophils; 7, Percent of neutrophils; 8, Percent of lymphocytes; 9, Percent of monocytes; 10, Percent of eosinophils, 11, Percent of basophils; 12, Red blood cells; 13, Hemoglobin; 14, Hematocrit; 15, Mean corpuscular volume, 16, Mean corpuscular hemoglobin; 17, Mean corpuscular hemoglobin concentration;

18, Red cell distribution width; 19, Platelets; 20, Mean platelet volume, S.D.: Standard Deviation, N: Number of animals; #, sample loss due to blood coagulation

Supplement 12. Hematological values for female rats after 1 month of recovery following 28-day oral administration of silver nanoparticles

|  |  | |  | |  | |  | |  | | |
| --- | --- | --- | --- | --- | --- | --- | --- | --- | --- | --- | --- |
| TESTS | | WBC^1^ | | NE^2^ | | LY^3^ | | MO^4^ | | EO^5^ | |
| UNITS | | K/μL | | K/μL | | K/μL | | K/μL | | K/μL | |
| GROUP: Control (0㎎/㎏) | | | | |  | |  | |  | | |
| MEAN | | 8.40 | | 2.12 | | 5.77 | | 0.47 | | 0.03 | |
| S.D. | | 1.60 | | 0.53 | | 1.17 | | 0.14 | | 0.04 | |
| N | | 10 | | 10 | | 10 | | 10 | | 10 | |
| GROUP: 10 nm (100㎎/㎏) | | | | |  | |  | |  | |  |
| MEAN | | 7.16 | | 1.70 | | 4.98 | | 0.45 | | 0.02 | |
| S.D. | | 1.73 | | 0.50 | | 1.39 | | 0.16 | | 0.02 | |
| N | | 5 | | 5 | | 5 | | 5 | | 5 | |
| GROUP: 10 nm (500㎎/㎏) | | | | |  | |  | |  | |  |
| MEAN | | 7.44 | | 1.88 | | 5.23 | | 0.31 | | 0.01 | |
| S.D. | | 1.15 | | 0.52 | | 1.01 | | 0.11 | | 0.01 | |
| N | | 5 | | 5 | | 5 | | 5 | | 5 | |
| GROUP: 25 nm (100㎎/㎏) | | | | |  | |  | |  | |  |
| MEAN | | 10.71 | | 2.95^＊^ | | 7.11 | | 0.61 | | 0.03 | |
| S.D. | | 2.08 | | 0.61 | | 1.47 | | 0.19 | | 0.02 | |
| N | | 5 | | 5 | | 5 | | 5 | | 5 | |
| GROUP: 25 nm (500㎎/㎏) | | | | |  | |  | |  | |  |
| MEAN | | 8.30 | | 2.21 | | 5.64 | | 0.42 | | 0.03 | |
| S.D. | | 3.20 | | 0.78 | | 2.64 | | 0.17 | | 0.05 | |
| N | | 5 | | 5 | | 5 | | 5 | | 5 | |

Continued

|  |  | |  | |  | |  | |  | | |
| --- | --- | --- | --- | --- | --- | --- | --- | --- | --- | --- | --- |
| TESTS | | BA^6^ | | NE^7^ | | LY^8^ | | MO^9^ | | EO^10^ | |
| UNITS | | K/μL | | % | | % | | % | | % | |
| GROUP: Control (0㎎/㎏) | | | | |  | |  | |  | | |
| MEAN | | 0.01 | | 25.21 | | 68.78 | | 5.61 | | 0.34 | |
| S.D. | | 0.01 | | 4.83 | | 5.50 | | 1.24 | | 0.42 | |
| N | | 10 | | 10 | | 10 | | 10 | | 10 | |
| GROUP: 10 nm (100㎎/㎏) | | | | |  | |  | |  | |  |
| MEAN | | 0.00 | | 24.13 | | 69.31 | | 6.21 | | 0.30 | |
| S.D. | | 0.00 | | 6.31 | | 6.95 | | 1.84 | | 0.15 | |
| N | | 5 | | 5 | | 5 | | 5 | | 5 | |
| GROUP: 10 nm (500㎎/㎏) | | | | |  | |  | |  | |  |
| MEAN | | 0.00 | | 25.44 | | 70.26 | | 4.14 | | 0.13 | |
| S.D. | | 0.00 | | 6.05 | | 6.36 | | 1.04 | | 0.12 | |
| N | | 5 | | 5 | | 5 | | 5 | | 5 | |
| GROUP: 25 nm (100㎎/㎏) | | | | |  | |  | |  | |  |
| MEAN | | 0.01 | | 27.78 | | 66.30 | | 5.62 | | 0.25 | |
| S.D. | | 0.01 | | 4.24 | | 3.16 | | 1.04 | | 0.18 | |
| N | | 5 | | 5 | | 5 | | 5 | | 5 | |
| GROUP: 25 nm (500㎎/㎏) | | | | |  | |  | |  | |  |
| MEAN | | 0.00 | | 27.22 | | 67.11 | | 5.24 | | 0.38 | |
| S.D. | | 0.01 | | 7.88 | | 9.67 | | 1.83 | | 0.63 | |
| N | | 5 | | 5 | | 5 | | 5 | | 5 | |

Continued

|  |  | |  | |  | |  | |  | | |
| --- | --- | --- | --- | --- | --- | --- | --- | --- | --- | --- | --- |
| TESTS | | BA^11^ | | RBC^12^ | | Hb^14^ | | HCT^14^ | | MCV^15^ | |
| UNITS | | % | | M/μL | | g/dL | | % | | fL | |
| GROUP: Control (0㎎/㎏) | | | | |  | |  | |  | | |
| MEAN | | 0.08 | | 7.22 | | 17.14 | | 36.57 | | 50.74 | |
| S.D. | | 0.13 | | 0.54 | | 1.16 | | 2.50 | | 1.81 | |
| N | | 10 | | 10 | | 10 | | 10 | | 10 | |
| GROUP: 10 nm (100㎎/㎏) | | | | |  | |  | |  | |  |
| MEAN | | 0.04 | | 7.10 | | 17.06 | | 36.32 | | 51.14 | |
| S.D. | | 0.05 | | 0.28 | | 0.92 | | 1.36 | | 1.02 | |
| N | | 5 | | 5 | | 5 | | 5 | | 5 | |
| GROUP: 25 nm (500㎎/㎏) | | | | |  | |  | |  | |  |
| MEAN | | 0.03 | | 7.11 | | 16.64 | | 35.22 | | 49.60 | |
| S.D. | | 0.02 | | 0.67 | | 1.23 | | 2.83 | | 1.57 | |
| N | | 5 | | 5 | | 5 | | 5 | | 5 | |
| GROUP: 25 nm (100㎎/㎏) | | | | |  | |  | |  | |  |
| MEAN | | 0.05 | | 7.00 | | 16.70 | | 36.34 | | 51.96 | |
| S.D. | | 0.07 | | 0.46 | | 0.87 | | 2.13 | | 2.64 | |
| N | | 5 | | 5 | | 5 | | 5 | | 5 | |
| GROUP: 25 nm (500㎎/㎏) | | | | |  | |  | |  | |  |
| MEAN | | 0.05 | | 7.16 | | 16.52 | | 36.50 | | 50.92 | |
| S.D. | | 0.08 | | 0.14 | | 0.73 | | 1.11 | | 1.35 | |
| N | | 5 | | 5 | | 5 | | 5 | | 5 | |

Continued

|  |  | |  | |  | |  | |  | | |
| --- | --- | --- | --- | --- | --- | --- | --- | --- | --- | --- | --- |
| TESTS | | MCH^16^ | | MCHC^17^ | | RDW^18^ | | PLT^19^ | | MPV^20^ | |
| UNITS | | pg | | g/dL | | % | | K/μL | | fL | |
| GROUP: Control (0㎎/㎏) | | | | |  | |  | |  | | |
| MEAN | | 23.79 | | 46.88 | | 16.36 | | 734.30 | | 14.19 | |
| S.D. | | 0.88 | | 1.11 | | 0.32 | | 132.29 | | 0.41 | |
| N | | 10 | | 10 | | 10 | | 10 | | 10 | |
| GROUP: 10 nm (100㎎/㎏) | | | | |  | |  | |  | |  |
| MEAN | | 24.02 | | 46.96 | | 16.14 | | 675.40 | | 4.14 | |
| S.D. | | 0.64 | | 1.29 | | 0.33 | | 44.49 | | 0.59 | |
| N | | 5 | | 5 | | 5 | | 5 | | 5 | |
| GROUP: 10 nm (500㎎/㎏) | | | | |  | |  | |  | |  |
| MEAN | | 23.44 | | 47.28 | | 16.62 | | 652.40 | | 4.46 | |
| S.D. | | 0.58 | | 1.14 | | 0.39 | | 91.86 | | 0.24 | |
| N | | 5 | | 5 | | 5 | | 5 | | 5 | |
| GROUP: 25 nm (100㎎/㎏) | | | | |  | |  | |  | |  |
| MEAN | | 23.88 | | 45.98 | | 16.28 | | 848.20 | | 4.12 | |
| S.D. | | 1.32 | | 0.92 | | 0.61 | | 150.58 | | 0.63 | |
| N | | 5 | | 5 | | 5 | | 5 | | 5 | |
| GROUP: 25 nm (500㎎/㎏) | | | | |  | |  | |  | |  |
| MEAN | | 23.04 | | 45.26* | | 16.10 | | 845.00 | | 3.74 | |
| S.D. | | 0.69 | | 1.02 | | 0.51 | | 83.91 | | 0.27 | |
| N | | 5 | | 5 | | 5 | | 5 | | 5 | |

^＊^: Significant different from control value, p ＜ 0.05

Supplement 13. Hematological values for female rats after 2 months of recovery following 28-day oral administration of silver nanoparticles

|  |  | |  | |  | |  | |  | | |
| --- | --- | --- | --- | --- | --- | --- | --- | --- | --- | --- | --- |
| TESTS | | WBC^1^ | | NE^2^ | | LY^3^ | | MO^4^ | | EO^5^ | |
| UNITS | | K/μL | | K/μL | | K/μL | | K/μL | | K/μL | |
| GROUP: Control (0㎎/㎏) | | | | |  | |  | |  | | |
| MEAN | | 7.69 | | 1.71 | | 5.67 | | 0.26 | | 0.05 | |
| S.D. | | 1.54 | | 0.63 | | 1.21 | | 0.11 | | 0.03 | |
| N | | 10 | | 10 | | 10 | | 10 | | 10 | |
| GROUP: 10 nm (100㎎/㎏) | | | | |  | |  | |  | |  |
| MEAN | | 9.14 | | 1.71 | | 7.09 | | 0.22 | | 0.09 | |
| S.D. | | 2.73 | | 0.76 | | 2.14 | | 0.07 | | 0.05 | |
| N | | 5 | | 5 | | 5 | | 5 | | 5 | |
| GROUP: 10 nm (500㎎/㎏) | | | | |  | |  | |  | |  |
| MEAN | | 10.65 | | 1.70 | | 8.55 | | 0.33 | | 0.06 | |
| S.D. | | 4.05 | | 0.64 | | 3.44 | | 0.12 | | 0.04 | |
| N | | 5 | | 5 | | 5 | | 5 | | 5 | |
| GROUP: 25 nm (100㎎/㎏) | | | | |  | |  | |  | |  |
| MEAN | | 7.19 | | 1.17 | | 5.80 | | 0.17 | | 0.05 | |
| S.D. | | 1.03 | | 0.34 | | 0.78 | | 0.06 | | 0.04 | |
| N | | 4 | | 4 | | 4 | | 4 | | 4 | |
| GROUP: 25 nm (500㎎/㎏) | | | | |  | |  | |  | |  |
| MEAN | | 7.23 | | 1.31 | | 5.57 | | 0.24 | | 0.09 | |
| S.D. | | 3.11 | | 1.03 | | 1.84 | | 0.12 | | 0.16 | |
| N# | | 4 | | 4 | | 4 | | 4 | | 4 | |

Continued

|  |  | |  | |  | |  | |  | | |
| --- | --- | --- | --- | --- | --- | --- | --- | --- | --- | --- | --- |
| TESTS | | BA^6^ | | NE^7^ | | LY^8^ | | MO^9^ | | EO^10^ | |
| UNITS | | K/μL | | % | | % | | % | | % | |
| GROUP: Control (0㎎/㎏) | | | | |  | |  | |  | | |
| MEAN | | 0.01 | | 22.11 | | 73.84 | | 3.33 | | 0.63 | |
| S.D. | | 0.01 | | 6.54 | | 7.34 | | 1.19 | | 0.32 | |
| N | | 10 | | 10 | | 10 | | 10 | | 10 | |
| GROUP: 10 nm (100㎎/㎏) | | | | |  | |  | |  | |  |
| MEAN | | 0.02 | | 18.36 | | 77.76 | | 2.68 | | 1.01 | |
| S.D. | | 0.04 | | 5.67 | | 5.03 | | 1.29 | | 0.52 | |
| N | | 5 | | 5 | | 5 | | 5 | | 5 | |
| GROUP: 10 nm (500㎎/㎏) | | | | |  | |  | |  | |  |
| MEAN | | 0.01 | | 16.43 | | 79.70 | | 3.16 | | 0.57 | |
| S.D. | | 0.02 | | 5.12 | | 5.88 | | 0.46 | | 0.58 | |
| N | | 5 | | 5 | | 5 | | 5 | | 5 | |
| GROUP: 25 nm (100㎎/㎏) | | | | |  | |  | |  | |  |
| MEAN | | 0.00 | | 16.09 | | 80.88 | | 2.34 | | 0.63 | |
| S.D. | | 0.01 | | 3.26 | | 3.52 | | 0.71 | | 0.42 | |
| N# | | 4 | | 4 | | 4 | | 4 | | 4 | |
| GROUP: 25 nm (500㎎/㎏) | | | | |  | |  | |  | |  |
| MEAN | | 0.04 | | 16.61 | | 78.97 | | 3.31 | | 0.80 | |
| S.D. | | 0.06 | | 5.69 | | 7.19 | | 0.81 | | 1.35 | |
| N# | | 4 | | 4 | | 4 | | 4 | | 4 | |

Continued

|  |  | |  | |  | |  | |  | | |
| --- | --- | --- | --- | --- | --- | --- | --- | --- | --- | --- | --- |
| TESTS | | BA^11^ | | RBC^12^ | | Hb^14^ | | HCT^14^ | | MCV^15^ | |
| UNITS | | % | | M/μL | | g/dL | | % | | fL | |
| GROUP: Control (0㎎/㎏) | | | | |  | |  | |  | | |
| MEAN | | 0.10 | | 8.01 | | 15.93 | | 38.36 | | 47.90 | |
| S.D. | | 0.09 | | 0.29 | | 0.52 | | 0.93 | | 1.25 | |
| N | | 10 | | 10 | | 10 | | 10 | | 10 | |
| GROUP: 10 nm (100㎎/㎏) | | | | |  | |  | |  | |  |
| MEAN | | 0.18 | | 7.13^＊^ | | 14.14^＊^ | | 34.48^＊^ | | 48.36 | |
| S.D. | | 0.39 | | 0.86 | | 2.20 | | 4.27 | | 1.39 | |
| N | | 5 | | 5 | | 5 | | 5 | | 5 | |
| GROUP: 10 nm (500㎎/㎏) | | | | |  | |  | |  | |  |
| MEAN | | 0.14 | | 7.95 | | 16.20 | | 38.80 | | 48.86 | |
| S.D. | | 0.31 | | 0.32 | | 0.86 | | 1.60 | | 1.79 | |
| N | | 5 | | 5 | | 5 | | 5 | | 5 | |
| GROUP: 25 nm (100㎎/㎏) | | | | |  | |  | |  | |  |
| MEAN | | 0.06 | | 7.63 | | 15.93 | | 37.43 | | 49.10 | |
| S.D. | | 0.09 | | 0.18 | | 1.06 | | 1.34 | | 1.91 | |
| N | | 4 | | 4 | | 4 | | 4 | | 4 | |
| GROUP: 25 nm (500㎎/㎏) | | | | |  | |  | |  | |  |
| MEAN | | 0.32 | | 7.52 | | 16.50 | | 37.13 | | 49.35 | |
| S.D. | | 0.53 | | 0.25 | | 0.70 | | 1.75 | | 1.22 | |
| N# | | 4 | | 4 | | 4 | | 4 | | 4 | |

Continued

|  |  | |  | |  | |  | |  | | |
| --- | --- | --- | --- | --- | --- | --- | --- | --- | --- | --- | --- |
| TESTS | | MCH^16^ | | MCHC^17^ | | RDW^18^ | | PLT^19^ | | MPV^20^ | |
| UNITS | | pg | | g/dL | | % | | K/μL | | fL | |
| GROUP: Control (0㎎/㎏) | | | | |  | |  | |  | | |
| MEAN | | 19.89 | | 41.55 | | 14.52 | | 1075.90 | | 6.92 | |
| S.D. | | 0.38 | | 1.03 | | 0.54 | | 169.70 | | 0.23 | |
| N | | 10 | | 10 | | 10 | | 10 | | 10 | |
| GROUP: 10 nm(100㎎/㎏) | | | | |  | |  | |  | |  |
| MEAN | | 19.76 | | 40.86 | | 14.26 | | 764.20 | | 6.74 | |
| S.D. | | 1.19 | | 1.57 | | 0.52 | | 262.19 | | 0.27 | |
| N | | 5 | | 5 | | 5 | | 5 | | 5 | |
| GROUP: 10 nm (500㎎/㎏) | | | | |  | |  | |  | |  |
| MEAN | | 20.40 | | 41.74 | | 14.44 | | 971.40 | | 6.70 | |
| S.D. | | 0.73 | | 0.99 | | 0.46 | | 245.70 | | 0.35 | |
| N | | 5 | | 5 | | 5 | | 5 | | 5 | |
| GROUP: 25 nm (100㎎/㎏) | | | | |  | |  | |  | |  |
| MEAN | | 20.88 | | 42.50 | | 14.25 | | 1052.25 | | 6.83 | |
| S.D. | | 1.17 | | 1.57 | | 0.34 | | 225.79 | | 0.33 | |
| N# | | 4 | | 4 | | 4 | | 4 | | 4 | |
| GROUP: 25 nm (500㎎/㎏) | | | | |  | |  | |  | |  |
| MEAN | | 21.93 | | 44.45** | | 14.50 | | 764.00* | | 6.60 | |
| S.D. | | 0.87 | | 1.24 | | 1.00 | | 84.01 | | 0.14 | |
| N# | | 4 | | 4 | | 4 | | 4 | | 4 | |

^＊^: Significant different from control value, p ＜ 0.05; ^＊＊^: Significant different from control value, p ＜ 0.01; #, sample loss due to blood coagulation

Supplement 14. Plasma coagulation values for male and female rats after 28-day oral administration of silver nanoparticles

| PLASMA COAGULATION VALUES | | | | | | | | |
| --- | --- | --- | --- | --- | --- | --- | --- | --- |
| STUDY: CU09-00140 | | |  | |  |  | PERIOD: 28 DAYS | |
| SEX: | | MALE | | |  | FEMALE | | |
| TESTS: | | PT | | APTT |  | PT | | APTT |
| UNITS: | | sec | | sec |  | Sec | | sec |
|  | | GROUP: CONTROL(0 ㎎/㎏) | | |  | GROUP: CONTROL(0 ㎎/㎏) | | |
| MEAN | | 15.51 | | 14.61 |  | 16.06 | | 16.80 |
| S.D. | | 0.47 | | 2.48 |  | 0.81 | | 5.13 |
| N# | | 9 | | 9 |  | 10 | | 10 |
|  |  | GROUP: 10 NM(100 ㎎/㎏) | | |  | GROUP: 10 NM(100 ㎎/㎏) | | |
| MEAN | | 14.30 | | 14.76 |  | 16.38 | | 14.20 |
| S.D. | | 1.35 | | 3.40 |  | 0.89 | | 3.12 |
| N | | 5 | | 5 |  | 5 | | 5 |
|  |  | GROUP: G3-1-0 (500 ㎎/㎏) | | |  | GROUP: G3-1-0 (500 ㎎/㎏) | | |
| MEAN | | 15.17 | | 15.67 |  | 16.56 | | 17.60 |
| S.D. | | 0.81 | | 3.66 |  | 0.38 | | 1.04 |
| N# | | 3 | | 3 |  | 5 | | 5 |
|  |  | GROUP: G2-2-0 (100 ㎎/㎏) | | |  | GROUP: G2-2-0 (100 ㎎/㎏) | | |
| MEAN | | 14.22 | | 12.98 |  | 16.28 | | 15.52 |
| S.D. | | 1.48 | | 1.91 |  | 0.91 | | 1.29 |
| N | | 5 | | 5 |  | 5 | | 5 |
|  |  | GROUP: G3-2-0 (500 ㎎/㎏) | | |  | GROUP: G3-2-0 (500 ㎎/㎏) | | |
| MEAN | | 15.30 | | 12.80 |  | 17.87* | | 15.97 |
| S.D. | | 0.55 | | 2.41 |  | 0.15 | | 2.50 |
| N# | | 4 | | 4 |  | 3 | | 3 |

PT: prothrombin time; APTT: active partial thromboplastin time; S.D.: Standard Deviation, N: Number of animals; ^＊^: Significant different from control value, p ＜ 0.05; #, sample loss due to blood coagulation

Supplement 15. Plasma coagulation values for male and female rats after 1 month of recovery following 28-day oral administration of silver nanoparticles

| PLASMA COAGULATION VALUES | | | | | | | | |
| --- | --- | --- | --- | --- | --- | --- | --- | --- |
| STUDY: CU09-00140 | | |  | |  |  | PERIOD: 57 DAYS | |
| SEX: | | MALE | | |  | FEMALE | | |
| TESTS: | | PT | | APTT |  | PT | | APTT |
| UNITS: | | sec | | sec |  | sec | | sec |
|  | | GROUP: G1-1-1 / G1-2-1 (0 ㎎/㎏) | | |  | GROUP: G1-1-1 / G1-2-1 (0 ㎎/㎏) | | |
| MEAN | | 17.14 | | 27.09 |  | 16.04 | | 37.42 |
| S.D. | | 2.89 | | 33.18 |  | 0.48 | | 46.54 |
| N | | 10 | | 9 |  | 10 | | 10 |
|  |  | GROUP: G2-1-1 (100 ㎎/㎏) | | |  | GROUP: G2-1-1 (100 ㎎/㎏) | | |
| MEAN | | 16.52 | | 14.46 |  | 15.90 | | 16.62 |
| S.D. | | 0.66 | | 1.88 |  | 0.67 | | 2.80 |
| N | | 5 | | 5 |  | 5 | | 5 |
|  |  | GROUP: G3-1-1 (500 ㎎/㎏) | | |  | GROUP: G3-1-1 (500 ㎎/㎏) | | |
| MEAN | | 17.20 | | 18.14 |  | 16.76 | | 25.05 |
| S.D. | | 1.72 | | 7.89 |  | 1.71 | | 18.85 |
| N | | 5 | | 5 |  | 5 | | 4 |
|  |  | GROUP: 2-2-1 (100 ㎎/㎏) | | |  | GROUP: G2-2-1 (100 ㎎/㎏) | | |
| MEAN | | 16.08 | | 15.12 |  | 16.02 | | 17.02 |
| S.D. | | 0.66 | | 2.07 |  | 0.28 | | 2.30 |
| N | | 5 | | 5 |  | 5 | | 5 |
|  |  | GROUP: 3-2-1 (500 ㎎/㎏) | | |  | GROUP: G3-2-1 (500 ㎎/㎏) | | |
| MEAN | | 16.28 | | 15.16 |  | 15.46 | | 13.92 |
| S.D. | | 0.46 | | 1.92 |  | 0.88 | | 2.28 |
| N | | 5 | | 5 |  | 5 | | 5 |

S.D.: Standard Deviation; N: Number of animals

Supplement 16. Plasma coagulation values for male and female rats after 2 months of recovery following 28-day oral administration of silver nanoparticles

| PLASMA COAGULATION VALUES | | | | | | | | |
| --- | --- | --- | --- | --- | --- | --- | --- | --- |
| STUDY: CU09-00140 | | |  | |  |  | PERIOD: 87 DAYS | |
| SEX: | | MALE | | |  | FEMALE | | |
| TESTS: | | PT | | APTT |  | PT | | APTT |
| UNITS: | | sec | | sec |  | sec | | sec |
|  | | GROUP: G1-1-2 / G1-2-2 (0 ㎎/㎏) | | |  | GROUP: G1-1-2 / G1-2-2 (0 ㎎/㎏) | | |
| MEAN | | 15.30 | | 12.95 |  | 15.78 | | 16.80 |
| S.D. | | 0.81 | | 2.20 |  | 0.72 | | 2.52 |
| N | | 10 | | 10 |  | 10 | | 10 |
|  |  | GROUP: G2-1-2 (100 ㎎/㎏) | | |  | GROUP: G2-1-2 (100 ㎎/㎏) | | |
| MEAN | | 15.20 | | 13.66 |  | 14.94 | | 12.74 |
| S.D. | | 1.04 | | 1.80 |  | 0.77 | | 3.51 |
| N | | 5 | | 5 |  | 5 | | 5 |
|  |  | GROUP: G3-1-2 (500 ㎎/㎏) | | |  | GROUP: G3-1-2 (500 ㎎/㎏) | | |
| MEAN | | 16.22 | | 14.26 |  | 15.28 | | 16.30 |
| S.D. | | 0.52 | | 1.25 |  | 1.43 | | 4.04 |
| N | | 5 | | 5 |  | 5 | | 5 |
|  |  | GROUP: G2-2-2 (100 ㎎/㎏) | | |  | GROUP: G2-2-2 (100 ㎎/㎏) | | |
| MEAN | | 15.00 | | 12.14 |  | 15.60 | | 16.78 |
| S.D. | | 0.68 | | 2.77 |  | 0.50 | | 2.58 |
| N | | 5 | | 5 |  | 4 | | 4 |
|  |  | GROUP: G3-2-2 (500 ㎎/㎏) | | |  | GROUP: G3-2-2 (500 ㎎/㎏) | | |
| MEAN | | 15.82 | | 13.22 |  | 15.38 | | 14.15 |
| S.D. | | 0.68 | | 1.37 |  | 1.67 | | 2.95 |
| N | | 5 | | 5 |  | 4 | | 4 |

S.D.: Standard Deviation; N: Number of animals

Supplement 17. Serum biochemical values for male rats after 28-day oral administration of silver nanoparticles

|  | | |  |  |  |  | |
| --- | --- | --- | --- | --- | --- | --- | --- |
| TESTS: | ALB^1^ | ALP^2^ | CA^3^ | CHO^4^ | CRE^5^ | GGT^6^ | GLU^7^ |
| UNITS: | g/dL | IU/L | mg/dL | mg/dL | mg/dL | IU/L | mg/dL |
| GROUP | CONTROL: 0 (㎎/㎏) | | |  |  |  |  |
| MEAN | 2.28 | 609.00 | 11.69 | 78.00 | 0.70 | 0.10 | 231.30 |
| S.D. | 0.08 | 132.97 | 0.57 | 12.88 | 0.05 | 0.32 | 34.82 |
| N | 10 | 10 | 10 | 10 | 10 | 10 | 10 |
| GROUP | 10 nm: 100 (㎎/㎏) | | |  |  |  |  |
| MEAN | 2.38 | 714.60 | 11.58 | 105.00^*^ | 0.68 | 0.00 | 205.60 |
| S.D. | 0.08 | 160.01 | 0.67 | 21.51 | 0.04 | 0.00 | 13.63 |
| N | 5 | 5 | 5 | 5 | 5 | 5 | 5 |
| GROUP | 10 nm: 500 (㎎/㎏) | | |  |  |  |  |
| MEAN | 2.40 | 796.67 | 11.20 | 99.67^*^ | 0.73 | 0.00 | 212.00 |
| S.D. | 0.00 | 85.87 | 0.44 | 10.12 | 0.06 | 0.00 | 15.72 |
| N | 3 | 3 | 3 | 3 | 3 | 3 | 3 |
| GROUP | 25 nm: 100 (㎎/㎏) | | |  |  |  |  |
| MEAN | 2.30 | 698.40 | 12.14 | 76.80* | 0.66 | 0.00 | 222.60 |
| S.D. | 0.12 | 174.34 | 0.57 | 18.98 | 0.05 | 0.00 | 30.05 |
| N | 5 | 5 | 5 | 5 | 5 | 5 | 5 |
| GROUP | 25 nm: 500 (㎎/㎏) | | |  |  |  |  |
| MEAN | 2.30 | 738.75 | 12.18 | 86.50 | 0.65 | 0.00 | 180.25 |
| S.D. | 0.00 | 135.08 | 0.45 | 8.81 | 0.13 | 0.00 | 20.07 |
| N# | 4 | 4 | 4 | 4 | 4 | 4 | 4 |

Continued

|  | | |  |  |  |  | |
| --- | --- | --- | --- | --- | --- | --- | --- |
| TESTS: | AST^8^ | ALT^9^ | LDH^10^ | MG^11^ | TP^12^ | UA^13^ | BUN^14^ |
| UNITS: | IU/L | IU/L | IU/L | mg/dL | G/dl | mg/dL | mg/dL |
| GROUP | CONTROL: 0 (㎎/㎏) | | |  |  |  |  |
| MEAN | 81.40 | 47.90 | 109.44 | 2.63 | 5.75 | 3.64 | 16.95 |
| S.D. | 15.46 | 12.44 | 39.57 | 0.30 | 0.22 | 1.72 | 2.02 |
| N | 10 | 10 | 9 | 10 | 10 | 10 | 10 |
| GROUP | 10 nm: 100 (㎎/㎏) | | |  |  |  |  |
| MEAN | 71.80 | 48.80 | 100.40 | 2.52 | 5.90 | 2.84 | 16.22 |
| S.D. | 4.15 | 7.40 | 15.42 | 0.33 | 0.34 | 1.07 | 2.85 |
| N | 5 | 5 | 5 | 5 | 5 | 5 | 5 |
| GROUP | 10 nm: 500 (㎎/㎏) | | |  |  |  |  |
| MEAN | 79.33 | 54.33 | 114.33 | 2.50 | 5.80 | 2.70 | 15.27 |
| S.D. | 10.12 | 1.15 | 30.09 | 0.36 | 0.10 | 1.01 | 2.44 |
| N# | 3 | 3 | 3 | 3 | 3 | 3 | 3 |
| GROUP | 25 nm: 100 (㎎/㎏) | | |  |  |  |  |
| MEAN | 81.60 | 47.20 | 120.80 | 2.68 | 5.78 | 3.22 | 15.60 |
| S.D. | 7.67 | 5.31 | 67.27 | 0.16 | 0.23 | 0.59 | 1.82 |
| N# | 5 | 5 | 5 | 5 | 5 | 5 | 5 |
| GROUP | 25 nm: 500 (㎎/㎏) | | |  |  |  |  |
| MEAN | 76.00 | 53.75 | 68.25 | 2.58 | 5.70 | 2.75 | 15.25 |
| S.D. | 4.32 | 10.44 | 4.50 | 0.15 | 0.08 | 0.48 | 2.80 |
| N | 4 | 4 | 4 | 4 | 4 | 4 | 4 |

Continued

|  | | |  |  |  |  | |
| --- | --- | --- | --- | --- | --- | --- | --- |
| TESTS: | T-BIL^15^ | IP^16^ | TG^17^ | CPK^18^ |  |  |  |
| UNITS: | mg/dL | mg/dL | mg/dL | U/L |  |  |  |
| GROUP | CONTROL: 0 (㎎/㎏) | | |  |  |  |  |
| MEAN | 0.04 | 11.21 | 59.80 | 207.70 |  |  |  |
| S.D. | 0.02 | 0.43 | 21.88 | 134.67 |  |  |  |
| N | 10 | 10 | 10 | 10 |  |  |  |
| GROUP | 10 nm: 100 (㎎/㎏) | | |  |  |  |  |
| MEAN | 0.04 | 11.24 | 55.00 | 146.40 |  |  |  |
| S.D. | 0.02 | 1.08 | 22.45 | 8.41 |  |  |  |
| N | 5 | 5 | 5 | 5 |  |  |  |
| GROUP | 10 nm: 500 (㎎/㎏) | | |  |  |  |  |
| MEAN | 0.03 | 10.93 | 63.00 | 167.33 |  |  |  |
| S.D. | 0.03 | 1.81 | 23.52 | 18.45 |  |  |  |
| N# | 3 | 3 | 3 | 3 |  |  |  |
| GROUP | 25 nm: 100 (㎎/㎏) | | |  |  |  |  |
| MEAN | 0.03 | 11.58 | 47.60 | 157.20 |  |  |  |
| S.D. | 0.02 | 0.91 | 6.77 | 34.27 |  |  |  |
| N | 5 | 5 | 5 | 5 |  |  |  |
| GROUP | 25 nm: 500 (㎎/㎏) | | |  |  |  |  |
| MEAN | 0.04 | 11.58 | 28.25 | 126.00 |  |  |  |
| S.D. | 0.01 | 0.31 | 15.33 | 15.71 |  |  |  |
| N# | 4 | 4 | 4 | 4 |  |  |  |

1, Albumin; 2, Alkaline phosphatase; 3, Calcium; 4, Total cholesterol; 5, Creatinine; 6, Gamma Glutamyl Transpeptidase; 7, Glucose; 8, Aspartate aminotransferase; 9, Alanine aminotransferase; 10, Lactate dehydrogenase; 11, Magnesium; 12, Total protein; 13, Uric acid; 14, Blood urea nitrogen; 15, Total bilirubin 16, Inorganic phosphorus; 17, Triglyceride; 18, Creatine phosphokinase, S.D.: Standard Deviation, N: Number of animals; ^＊^: Significant different from control value, p ＜ 0.05, ; #, sample loss due to blood coagulation

Supplement 18. Serum biochemical values for male rats after 1 month of recovery following 28-day oral administration of silver nanoparticles

|  | | |  |  |  |  | |
| --- | --- | --- | --- | --- | --- | --- | --- |
| TESTS: | ALB^1^ | ALP^2^ | CA^3^ | CHO^4^ | CRE^5^ | GGT^6^ | GLU^7^ |
| UNITS: | g/dL | IU/L | mg/dL | mg/dL | mg/dL | IU/L | mg/dL |
| GROUP | Control: 0 (㎎/㎏) | | |  |  |  |  |
| MEAN | 2.31 | 334.00 | 11.16 | 82.70 | 0.75 | 0.00 | 188.70 |
| S.D. | 0.12 | 77.92 | 0.36 | 16.54 | 0.08 | 0.00 | 49.25 |
| N | 10 | 10 | 10 | 10 | 10 | 10 | 10 |
| GROUP | 10 nm: 100 (㎎/㎏) | | |  |  |  |  |
| MEAN | 2.26 | 367.20 | 11.26 | 73.80 | 0.72 | 0.00 | 204.60 |
| S.D. | 0.17 | 57.71 | 0.54 | 9.98 | 0.08 | 0.00 | 56.76 |
| N | 5 | 5 | 5 | 5 | 5 | 5 | 5 |
| GROUP | 10 nm: 500 (㎎/㎏) | | |  |  |  |  |
| MEAN | 2.36 | 380.00 | 11.46 | 75.80 | 0.74 | 0.00 | 181.60 |
| S.D. | 0.11 | 116.47 | 0.62 | 13.95 | 0.09 | 0.00 | 43.65 |
| N | 5 | 5 | 5 | 5 | 5 | 5 | 5 |
| GROUP | 25 nm: 100 (㎎/㎏) | | |  |  |  |  |
| MEAN | 2.34 | 314.80 | 11.48 | 74.80 | 0.72 | 0.00 | 211.40 |
| S.D. | 0.11 | 69.35 | 0.31 | 13.31 | 0.08 | 0.00 | 40.86 |
| N | 5 | 5 | 5 | 5 | 5 | 5 | 5 |
| GROUP | 25 nm: 500 (㎎/㎏) | | |  |  |  |  |
| MEAN | 2.32 | 370.00 | 11.26 | 82.40 | 0.80 | 0.00 | 207.80 |
| S.D. | 0.16 | 102.96 | 0.52 | 18.56 | 0.07 | 0.00 | 55.01 |
| N | 5 | 5 | 5 | 5 | 5 | 5 | 5 |

Continued

|  | | |  |  |  |  | |
| --- | --- | --- | --- | --- | --- | --- | --- |
| TESTS: | AST^8^ | ALT^9^ | LDH^10^ | MG^11^ | TP^12^ | UA^13^ | BUN^14^ |
| UNITS: | IU/L | IU/L | IU/L | mg/dL | G/dl | mg/dL | mg/dL |
| GROUP | Control: 0 (㎎/㎏) | | |  |  |  |  |
| MEAN | 72.00 | 38.30 | 136.00 | 2.29 | 5.93 | 2.55 | 15.07 |
| S.D. | 9.40 | 6.31 | 133.01 | 0.39 | 0.32 | 1.76 | 2.99 |
| N | 10 | 10 | 10 | 10 | 10 | 10 | 10 |
| GROUP | 10 nm: 100 (㎎/㎏) | | |  |  |  |  |
| MEAN | 82.60 | 42.20 | 134.60 | 2.56 | 5.76 | 3.02 | 17.22 |
| S.D. | 10.06 | 10.47 | 66.00 | 0.35 | 0.34 | 2.16 | 2.14 |
| N | 5 | 5 | 5 | 5 | 5 | 5 | 5 |
| GROUP | 10 nm: 500 (㎎/㎏) | | |  |  |  |  |
| MEAN | 81.40 | 43.20 | 157.40 | 2.28 | 5.96 | 2.24 | 16.36 |
| S.D. | 9.86 | 8.32 | 138.52 | 0.18 | 0.33 | 0.68 | 2.80 |
| N | 5 | 5 | 5 | 5 | 5 | 5 | 5 |
| GROUP | 25 nm: 100 (㎎/㎏) | | |  |  |  |  |
| MEAN | 82.20 | 42.20 | 181.80 | 2.40 | 5.96 | 3.54 | 13.80 |
| S.D. | 12.81 | 9.86 | 174.55 | 0.20 | 0.23 | 1.48 | 2.58 |
| N | 5 | 5 | 5 | 5 | 5 | 5 | 5 |
| GROUP | 25 nm: 500 (㎎/㎏) | | |  |  |  |  |
| MEAN | 82.60 | 42.80 | 197.80 | 2.32 | 5.82 | 2.70 | 14.14 |
| S.D. | 12.48 | 8.17 | 158.28 | 0.13 | 0.39 | 0.91 | 1.13 |
| N | 5 | 5 | 5 | 5 | 5 | 5 | 5 |

Continued

|  | | |  |  |  |  | |
| --- | --- | --- | --- | --- | --- | --- | --- |
| TESTS: | T-BIL^15^ | IP^16^ | TG^17^ | CPK^18^ |  |  |  |
| UNITS: | mg/dL | mg/dL | mg/dL | U/L |  |  |  |
| GROUP | Control: 0 (㎎/㎏) | | |  |  |  |  |
| MEAN | 0.07 | 9.47 | 59.10 | 139.80 |  |  |  |
| S.D. | 0.03 | 1.18 | 21.29 | 89.13 |  |  |  |
| N | 10 | 10 | 10 | 10 |  |  |  |
| GROUP | 10 nm: 100 (㎎/㎏) | | |  |  |  |  |
| MEAN | 0.08 | 9.72 | 45.00 | 145.80 |  |  |  |
| S.D. | 0.04 | 1.03 | 18.85 | 44.23 |  |  |  |
| N | 5 | 5 | 5 | 5 |  |  |  |
| GROUP | 10 nm: 500 (㎎/㎏) | | |  |  |  |  |
| MEAN | 0.09 | 8.90 | 45.80 | 139.60 |  |  |  |
| S.D. | 0.03 | 0.85 | 28.61 | 49.59 |  |  |  |
| N | 5 | 5 | 5 | 5 |  |  |  |
| GROUP | 25 nm: 100 (㎎/㎏) | | |  |  |  |  |
| MEAN | 0.06 | 9.56 | 68.80 | 166.00 |  |  |  |
| S.D. | 0.02 | 0.48 | 44.94 | 85.79 |  |  |  |
| N | 5 | 5 | 5 | 5 |  |  |  |
| GROUP | 25 nm: 500 (㎎/㎏) | | |  |  |  |  |
| MEAN | 0.07 | 9.12 | 62.20 | 174.00 |  |  |  |
| S.D. | 0.04 | 0.25 | 26.57 | 62.80 |  |  |  |
| N | 5 | 5 | 5 | 5 |  |  |  |

1, Albumin; 2, Alkaline phosphatase; 3, Calcium; 4, Total cholesterol; 5, Creatinine; 6, Gamma Glutamyl Transpeptidase; 7, Glucose;

8, Aspartate aminotransferase; 9, Alanine aminotransferase; 10, Lactate dehydrogenase; 11, Magnesium; 12, Total protein; 13, Uric acid; 14, Blood urea nitrogen, 15, Total bilirubin 16, Inorganic phosphorus; 17, Triglyceride; 18, Creatine phosphokinase S.D.: Standard Deviation

N: Number of animals

Supplement 19. Serum biochemical values for male rats after 2 months of recovery following 28-day oral administration of silver nanoparticles

|  | | |  |  |  |  | |
| --- | --- | --- | --- | --- | --- | --- | --- |
| TESTS: | ALB^1^ | ALP^2^ | CA^3^ | CHO^4^ | CRE^5^ | GGT^6^ | GLU^7^ |
| UNITS: | g/dL | IU/L | mg/dL | mg/dL | mg/dL | IU/L | mg/dL |
| GROUP | Control: 0 (㎎/㎏) | | |  |  |  |  |
| MEAN | 2.35 | 213.30 | 10.60 | 86.60 | 0.63 | 0.10 | 187.90 |
| S.D. | 0.10 | 38.30 | 0.31 | 17.73 | 0.07 | 0.32 | 37.69 |
| N | 10 | 10 | 10 | 10 | 10 | 10 | 10 |
| GROUP | 10 nm: 100 (㎎/㎏) | | |  |  |  |  |
| MEAN | 2.46 | 240.80 | 11.06 | 88.40 | 0.62 | 0.00 | 184.60 |
| S.D. | 0.15 | 87.16 | 0.32 | 20.78 | 0.04 | 0.00 | 30.20 |
| N | 5 | 5 | 5 | 5 | 5 | 5 | 5 |
| GROUP | 10 nm: 500 (㎎/㎏) | | |  |  |  |  |
| MEAN | 2.34 | 262.00 | 10.60 | 91.80 | 0.56 | 0.00 | 165.40 |
| S.D. | 0.11 | 84.30 | 0.19 | 13.22 | 0.09 | 0.00 | 30.29 |
| N | 5 | 5 | 5 | 5 | 5 | 5 | 5 |
| GROUP | 25 nm: 100 (㎎/㎏) | | |  |  |  |  |
| MEAN | 2.34 | 215.60 | 10.72 | 81.40 | 0.74^＊＊^ | 0.20 | 196.20 |
| S.D. | 0.09 | 43.64 | 0.50 | 29.92 | 0.09 | 0.45 | 42.42 |
| N | 5 | 5 | 5 | 5 | 5 | 5 | 5 |
| GROUP | 25 nm: 500 (㎎/㎏) | | |  |  |  |  |
| MEAN | 2.36 | 276.40 | 10.56 | 79.60 | 1.00^＊＊^ | 0.40 | 203.80 |
| S.D. | 0.05 | 89.73 | 0.34 | 23.86 | 0.07 | 0.55 | 8.32 |
| N | 5 | 5 | 5 | 5 | 5 | 5 | 5 |

Continued

| STUDY ID: CU09-00140 | | |  |  |  | SEX: MALE | |
| --- | --- | --- | --- | --- | --- | --- | --- |
| TESTS: | AST^8^ | ALT^9^ | LDH^10^ | MG^11^ | TP^12^ | UA^13^ | BUN^14^ |
| UNITS: | IU/L | IU/L | IU/L | mg/dL | G/dl | mg/dL | mg/dL |
| GROUP | Control: 0 (㎎/㎏) | | |  |  |  |  |
| MEAN | 77.60 | 34.70 | 220.40 | 2.22 | 6.05 | 2.10 | 14.07 |
| S.D. | 7.14 | 4.92 | 226.52 | 0.36 | 0.24 | 0.68 | 2.51 |
| N | 10 | 10 | 10 | 10 | 10 | 10 | 10 |
| GROUP | 10 nm: 100 (㎎/㎏) | | |  |  |  |  |
| MEAN | 80.80 | 43.00 | 98.20 | 2.32 | 6.14 | 2.36 | 12.32 |
| S.D. | 14.92 | 5.43 | 23.44 | 0.23 | 0.49 | 0.30 | 0.88 |
| N | 5 | 5 | 5 | 5 | 5 | 5 | 5 |
| GROUP | 10 nm: 500 (㎎/㎏) | | |  |  |  |  |
| MEAN | 77.00 | 42.40 | 80.60 | 2.22 | 6.14 | 1.66 | 13.54 |
| S.D. | 9.97 | 5.03 | 25.58 | 0.26 | 0.24 | 0.59 | 1.77 |
| N | 5 | 5 | 5 | 5 | 5 | 5 | 5 |
| GROUP | 25 nm: 100 (㎎/㎏) | | |  |  |  |  |
| MEAN | 81.60 | 35.60 | 198.60 | 2.42 | 5.96 | 3.16** | 12.48 |
| S.D. | 8.17 | 4.77 | 90.40 | 0.34 | 0.29 | 1.22 | 1.81 |
| N | 5 | 5 | 5 | 5 | 5 | 5 | 5 |
| GROUP | 25 nm: 500 (㎎/㎏) | | |  |  |  |  |
| MEAN | 82.80 | 51.80 | 170.20 | 2.48 | 6.00 | 3.20** | 12.98 |
| S.D. | 7.26 | 27.20 | 145.18 | 0.31 | 0.37 | 0.55 | 1.23 |
| N | 5 | 5 | 5 | 5 | 5 | 5 | 5 |

Continued

|  | | |  |  |  |  | |
| --- | --- | --- | --- | --- | --- | --- | --- |
| TESTS: | T-BIL^15^ | IP^16^ | TG^17^ | CPK^18^ |  |  |  |
| UNITS: | mg/dL | mg/dL | mg/dL | U/L |  |  |  |
| GROUP | Control: 0 (㎎/㎏) | | |  |  |  |  |
| MEAN | 0.06 | 6.82 | 65.50 | 154.40 |  |  |  |
| S.D. | 0.02 | 0.33 | 28.00 | 85.27 |  |  |  |
| N | 10 | 10 | 10 | 10 |  |  |  |
| GROUP | 10 nm: 100 (㎎/㎏) | | |  |  |  |  |
| MEAN | 0.08 | 6.38 | 87.80 | 126.20 |  |  |  |
| S.D. | 0.03 | 0.19 | 45.19 | 26.45 |  |  |  |
| N | 5 | 5 | 5 | 5 |  |  |  |
| GROUP | 10 nm: 500 (㎎/㎏) | | |  |  |  |  |
| MEAN | 0.08 | 6.78 | 55.80 | 102.60 |  |  |  |
| S.D. | 0.03 | 0.57 | 28.76 | 9.79 |  |  |  |
| N | 5 | 5 | 5 | 5 |  |  |  |
| GROUP | 25 nm: 100 (㎎/㎏) | | |  |  |  |  |
| MEAN | 0.06 | 6.86 | 66.20 | 147.00 |  |  |  |
| S.D. | 0.01 | 0.40 | 10.03 | 35.76 |  |  |  |
| N | 5 | 5 | 5 | 5 |  |  |  |
| GROUP | 25 nm: 500 (㎎/㎏) | | |  |  |  |  |
| MEAN | 0.04 | 10.04** | 57.20 | 125.80 |  |  |  |
| S.D. | 0.03 | 0.11 | 21.18 | 55.24 |  |  |  |
| N | 5 | 5 | 5 | 5 |  |  |  |

1, Albumin; 2, Alkaline phosphatase; 3, Calcium; 4, Total cholesterol; 5, Creatinine; 6, Gamma Glutamyl Transpeptidase; 7, Glucose;

8, Aspartate aminotransferase; 9, Alanine aminotransferase; 10, Lactate dehydrogenase; 11, Magnesium; 12, Total protein; 13, Uric acid; 14, 15, Total bilirubin 16, Inorganic phosphorus; 17, Triglyceride; 18, Creatine phosphokinase, Blood urea nitrogen, S.D.: Standard Deviation, N: Number of animals, ^＊＊^: Significant different from control value, p ＜ 0.01

Supplement 20. Serum biochemical values for female rats after 28-day oral administration of silver nanoparticles

| STUDY ID: CU09-00140 | | |  |  |  | SEX: FEMALE | |
| --- | --- | --- | --- | --- | --- | --- | --- |
| TESTS: | ALB^1^ | ALP^2^ | CA^3^ | CHO^4^ | CRE^5^ | GGT^6^ | GLU^7^ |
| UNITS: | g/dL | IU/L | mg/dL | mg/dL | mg/dL | IU/L | mg/dL |
| GROUP | CONTROL: 0 (㎎/㎏) | | |  |  |  |  |
| MEAN | 2.61 | 388.70 | 11.63 | 92.60 | 0.77 | 0.10 | 199.20 |
| S.D. | 0.17 | 107.13 | 0.54 | 15.54 | 0.09 | 0.32 | 25.38 |
| N | 10 | 10 | 10 | 10 | 10 | 10 | 10 |
| GROUP | 10 nm: 100 (㎎/㎏) | | |  |  |  |  |
| MEAN | 2.54 | 555.20** | 11.46 | 115.00 | 0.74 | 0.20 | 217.20 |
| S.D. | 0.05 | 83.52 | 0.43 | 15.65 | 0.05 | 0.45 | 20.39 |
| N | 5 | 5 | 5 | 5 | 5 | 5 | 5 |
| GROUP | 10 nm: 500 (㎎/㎏) | | |  |  |  |  |
| MEAN | 2.50 | 534.40** | 11.14 | 101.00 | 0.80 | 0.20 | 194.80 |
| S.D. | 0.07 | 62.66 | 0.36 | 12.43 | 0.12 | 0.45 | 30.26 |
| N | 5 | 5 | 5 | 5 | 5 | 5 | 5 |
| GROUP | 25 nm: 100 (㎎/㎏) | | |  |  |  |  |
| MEAN | 2.54 | 445.20 | 12.54 | 104.40 | 0.64 | 0.20 | 198.20 |
| S.D. | 0.23 | 38.45 | 1.77 | 14.59 | 0.09 | 0.45 | 32.82 |
| N | 5 | 5 | 5 | 5 | 5 | 5 | 5 |
| GROUP | 25 nm: 500 (㎎/㎏) | | |  |  |  |  |
| MEAN | 2.64 | 469.00 | 12.46 | 99.20 | 0.86 | 0.00 | 195.20 |
| S.D. | 0.38 | 79.85 | 1.31 | 12.76 | 0.17 | 0.00 | 65.12 |
| N | 5 | 5 | 5 | 5 | 5 | 5 | 5 |

Continued

|  | | |  |  |  |  | |
| --- | --- | --- | --- | --- | --- | --- | --- |
| TESTS: | AST^8^ | ALT^9^ | LDH^10^ | MG^11^ | TP^12^ | UA^13^ | BUN^14^ |
| UNITS: | IU/L | IU/L | IU/L | mg/dL | G/dl | mg/dL | mg/dL |
| GROUP | CONTROL: 0 (㎎/㎏) | | |  |  |  |  |
| MEAN | 71.10 | 44.40 | 69.90 | 2.76 | 6.12 | 2.66 | 17.83 |
| S.D. | 8.03 | 9.00 | 19.12 | 0.20 | 0.27 | 0.41 | 4.51 |
| N | 10 | 10 | 10 | 10 | 10 | 10 | 10 |
| GROUP | 10 nm: 100 (㎎/㎏) | | |  |  |  |  |
| MEAN | 77.60 | 49.20 | 72.60 | 2.82 | 6.02 | 2.64 | 23.62 |
| S.D. | 7.16 | 8.35 | 23.54 | 0.13 | 0.11 | 0.53 | 4.23 |
| N | 5 | 5 | 5 | 5 | 5 | 5 | 5 |
| GROUP | 10 nm: 500 (㎎/㎏) | | |  |  |  |  |
| MEAN | 75.80 | 51.40 | 117.60 | 2.66 | 6.02 | 2.18 | 19.64 |
| S.D. | 5.63 | 7.70 | 49.30 | 0.18 | 0.22 | 0.40 | 4.72 |
| N | 5 | 5 | 5 | 5 | 5 | 5 | 5 |
| GROUP | 25 nm: 100 (㎎/㎏) | | |  |  |  |  |
| MEAN | 80.00 | 47.60 | 149.80 | 2.80 | 6.22 | 2.46 | 17.04 |
| S.D. | 6.52 | 6.95 | 112.65 | 0.23 | 0.33 | 0.80 | 2.17 |
| N | 5 | 5 | 5 | 5 | 5 | 5 | 5 |
| GROUP | 25 nm: 500 (㎎/㎏) | | |  |  |  |  |
| MEAN | 86.20* | 46.20 | 236.40 | 2.82 | 5.96 | 2.04 | 18.20 |
| S.D. | 14.04 | 6.76 | 207.41 | 0.24 | 0.21 | 0.79 | 3.49 |
| N | 5 | 5 | 5 | 5 | 5 | 5 | 5 |

Continued

|  | | |  |  |  |  | |
| --- | --- | --- | --- | --- | --- | --- | --- |
| TESTS: | T-BIL^15^ | IP^16^ | TG^17^ | CPK^18^ |  |  |  |
| UNITS: | mg/dL | mg/dL | mg/dL | U/L |  |  |  |
| GROUP | CONTROL: 0 (㎎/㎏) | | |  |  |  |  |
| MEAN | 0.05 | 10.49 | 40.20 | 119.30 |  |  |  |
| S.D. | 0.03 | 0.71 | 18.24 | 19.67 |  |  |  |
| N | 10 | 10 | 10 | 10 |  |  |  |
| GROUP | 10 nm: 100 (㎎/㎏) | | |  |  |  |  |
| MEAN | 0.04 | 10.24 | 32.60 | 113.80 |  |  |  |
| S.D. | 0.02 | 1.70 | 7.16 | 24.79 |  |  |  |
| N | 5 | 5 | 5 | 5 |  |  |  |
| GROUP | 10 nm: 500 (㎎/㎏) | | |  |  |  |  |
| MEAN | 0.05 | 9.68 | 22.60 | 128.20 |  |  |  |
| S.D. | 0.02 | 0.48 | 10.24 | 33.55 |  |  |  |
| N | 5 | 5 | 5 | 5 |  |  |  |
| GROUP | 25 nm: 100 (㎎/㎏) | | |  |  |  |  |
| MEAN | 0.04 | 10.18 | 64.60 | 150.80 |  |  |  |
| S.D. | 0.03 | 1.04 | 68.37 | 49.10 |  |  |  |
| N | 5 | 5 | 5 | 5 |  |  |  |
| GROUP | 25 nm: 500 (㎎/㎏) | | |  |  |  |  |
| MEAN | 0.04 | 9.92 | 25.60 | 199.40 |  |  |  |
| S.D. | 0.02 | 1.49 | 7.37 | 89.67 |  |  |  |
| N | 5 | 5 | 5 | 5 |  |  |  |

1, Albumin; 2, Alkaline phosphatase; 3, Calcium; 4, Total cholesterol; 5, Creatinine; 6, Gamma Glutamyl Transpeptidase; 7, Glucose;

8, Aspartate aminotransferase; 9, Alanine aminotransferase; 10, Lactate dehydrogenase; 11, Magnesium; 12, Total protein; 13, Uric acid; 14, Blood urea nitrogen, 15, Total bilirubin 16, Inorganic phosphorus; 17, Triglyceride; 18, Creatine phosphokinase S.D.: Standard Deviation

N: Number of animals, ^＊^: Significant different from control value, p ＜ 0.05; ^＊＊^: Significant different from control value, p ＜ 0.01

Supplement 21. Serum biochemical values for female rats after 1 month of recovery following 28-day oral administration of silver nanoparticles

|  | | |  |  |  |  | |
| --- | --- | --- | --- | --- | --- | --- | --- |
| TESTS: | ALB^1^ | ALP^2^ | CA^3^ | CHO^4^ | CRE^5^ | GGT^6^ | GLU^7^ |
| UNITS: | g/dL | IU/L | mg/dL | mg/dL | mg/dL | IU/L | mg/dL |
| GROUP | Control: 0 (㎎/㎏) | | |  |  |  |  |
| MEAN | 2.70 | 187.60 | 11.04 | 93.40 | 0.79 | 0.90 | 157.70 |
| S.D. | 0.23 | 35.29 | 0.25 | 12.84 | 0.09 | 2.18 | 21.49 |
| N | 10 | 10 | 10 | 10 | 10 | 10 | 10 |
| GROUP | 10 nm: 100 (㎎/㎏) | | |  |  |  |  |
| MEAN | 2.84 | 278.40** | 11.10 | 92.80 | 0.84 | 0.20 | 178.60 |
| S.D. | 0.19 | 25.67 | 0.17 | 15.74 | 0.05 | 0.45 | 56.55 |
| N | 5 | 5 | 5 | 5 | 5 | 5 | 5 |
| GROUP | 10 nm: 500 (㎎/㎏) | | |  |  |  |  |
| MEAN | 2.86 | 229.40 | 11.04 | 86.60 | 0.74 | 0.20 | 164.00 |
| S.D. | 0.15 | 72.59 | 0.19 | 15.66 | 0.11 | 0.45 | 57.71 |
| N | 5 | 5 | 5 | 5 | 5 | 5 | 5 |
| GROUP | 25 nm: 100 (㎎/㎏) | | |  |  |  |  |
| MEAN | 2.74 | 137.20 | 11.08 | 84.80 | 0.68 | 0.00 | 123.80 |
| S.D. | 0.18 | 24.79 | 0.49 | 14.34 | 0.13 | 0.00 | 16.35 |
| N | 5 | 5 | 5 | 5 | 5 | 5 | 5 |
| GROUP | 25 nm: 500 (㎎/㎏) | | |  |  |  |  |
| MEAN | 2.62 | 208.80 | 11.00 | 88.20 | 0.72 | 0.00 | 141.60 |
| S.D. | 0.23 | 72.96 | 0.37 | 22.96 | 0.08 | 0.00 | 31.07 |
| N | 5 | 5 | 5 | 5 | 5 | 5 | 5 |

Continued

|  | | |  |  |  |  | |
| --- | --- | --- | --- | --- | --- | --- | --- |
| TESTS: | AST^8^ | ALT^9^ | LDH^10^ | MG^11^ | TP^12^ | UA^13^ | BUN^14^ |
| UNITS: | IU/L | IU/L | IU/L | mg/dL | G/dl | mg/dL | mg/dL |
| GROUP | Control: 0 (㎎/㎏) | | |  |  |  |  |
| MEAN | 81.30 | 38.10 | 107.50 | 2.32 | 6.16 | 1.50 | 14.38 |
| S.D. | 17.90 | 9.41 | 134.30 | 0.23 | 0.24 | 0.49 | 2.53 |
| N | 10 | 10 | 10 | 10 | 10 | 10 | 10 |
| GROUP | 10 nm: 100 (㎎/㎏) | | |  |  |  |  |
| MEAN | 81.80 | 42.20 | 92.00 | 2.60 | 6.32 | 1.80 | 17.20 |
| S.D. | 6.76 | 10.57 | 85.77 | 0.19 | 0.41 | 0.96 | 2.97 |
| N | 5 | 5 | 5 | 5 | 5 | 5 | 5 |
| GROUP | 10 nm: 500 (㎎/㎏) | | |  |  |  |  |
| MEAN | 74.60 | 30.60 | 53.80 | 2.52 | 6.30 | 1.70 | 17.22 |
| S.D. | 8.38 | 1.14 | 9.68 | 0.41 | 0.24 | 0.86 | 6.07 |
| N | 5 | 5 | 5 | 5 | 5 | 5 | 5 |
| GROUP | 25 nm: 100 (㎎/㎏) | | |  |  |  |  |
| MEAN | 77.20 | 33.80 | 60.40 | 2.34 | 6.30 | 1.74 | 15.08 |
| S.D. | 12.79 | 6.06 | 27.35 | 0.55 | 0.40 | 1.72 | 2.41 |
| N | 5 | 5 | 5 | 5 | 5 | 5 | 5 |
| GROUP | 25 nm: 500 (㎎/㎏) | | |  |  |  |  |
| MEAN | 81.40 | 38.40 | 94.80 | 2.32 | 6.12 | 1.42 | 16.62 |
| S.D. | 14.91 | 7.30 | 53.33 | 0.27 | 0.56 | 0.73 | 3.93 |
| N | 5 | 5 | 5 | 5 | 5 | 5 | 5 |

Continued

|  | | |  |  |  |  | |
| --- | --- | --- | --- | --- | --- | --- | --- |
| TESTS: | T-BIL^15^ | IP^16^ | TG^17^ | CPK^18^ |  |  |  |
| UNITS: | mg/dL | mg/dL | mg/dL | U/L |  |  |  |
| GROUP | Control: 0 (㎎/㎏) | | |  |  |  |  |
| MEAN | 0.07 | 8.14 | 52.60 | 116.40 |  |  |  |
| S.D. | 0.03 | 0.63 | 24.27 | 92.60 |  |  |  |
| N | 10 | 10 | 10 | 10 |  |  |  |
| GROUP | 10 nm: 100 (㎎/㎏) | | |  |  |  |  |
| MEAN | 0.09 | 7.76 | 26.20 | 95.40 |  |  |  |
| S.D. | 0.04 | 0.55 | 8.44 | 38.78 |  |  |  |
| N | 5 | 5 | 5 | 5 |  |  |  |
| GROUP | 10 nm: 500 (㎎/㎏) | | |  |  |  |  |
| MEAN | 0.08 | 9.06 | 39.80 | 90.20 |  |  |  |
| S.D. | 0.02 | 2.09 | 15.02 | 12.15 |  |  |  |
| N | 5 | 5 | 5 | 5 |  |  |  |
| GROUP | 25 nm: 100 (㎎/㎏) | | |  |  |  |  |
| MEAN | 0.09 | 8.46 | 29.40 | 86.80 |  |  |  |
| S.D. | 0.03 | 1.10 | 2.07 | 18.32 |  |  |  |
| N | 5 | 5 | 5 | 5 |  |  |  |
| GROUP | 25 nm: 500 (㎎/㎏) | | |  |  |  |  |
| MEAN | 0.07 | 8.16 | 34.40 | 107.20 |  |  |  |
| S.D. | 0.02 | 0.76 | 16.70 | 29.64 |  |  |  |
| N | 5 | 5 | 5 | 5 |  |  |  |

1, Albumin; 2, Alkaline phosphatase; 3, Calcium; 4, Total cholesterol; 5, Creatinine; 6, Gamma Glutamyl Transpeptidase; 7, Glucose;

8, Aspartate aminotransferase; 9, Alanine aminotransferase; 10, Lactate dehydrogenase; 11, Magnesium; 12, Total protein; 13, Uric acid; 14, Blood urea nitrogen; 15, Total bilirubin 16, Inorganic phosphorus; 17, Triglyceride; 18, Creatine phosphokinase; S.D.: Standard Deviation

N: Number of animals; ^＊＊^: Significant different from control value, p ＜ 0.01

Supplement 22. Serum biochemical values for female rats after 2 months of recovery following 28-day oral administration of silver nanoparticles

|  | | |  |  |  |  | |
| --- | --- | --- | --- | --- | --- | --- | --- |
| TESTS: | ALB^1^ | ALP^2^ | CA^3^ | CHO^4^ | CRE^5^ | GGT^6^ | GLU^7^ |
| UNITS: | g/dL | IU/L | mg/dL | mg/dL | mg/dL | IU/L | mg/dL |
| GROUP | Control: 0 (㎎/㎏) | | |  |  |  |  |
| MEAN | 2.80 | 167.50 | 10.62 | 100.10 | 1.08 | 0.40 | 153.80 |
| S.D. | 0.21 | 63.52 | 0.35 | 12.84 | 0.08 | 0.52 | 19.24 |
| N | 10 | 10 | 10 | 10 | 10 | 10 | 10 |
| GROUP | 10 nm: 100 (㎎/㎏) | | |  |  |  |  |
| MEAN | 2.76 | 161.60 | 10.64 | 88.60 | 1.06 | 0.40 | 165.60 |
| S.D. | 0.22 | 45.11 | 0.38 | 9.29 | 0.15 | 0.55 | 31.75 |
| N | 5 | 5 | 5 | 5 | 5 | 5 | 5 |
| GROUP | 10 nm: 500 (㎎/㎏) | | |  |  |  |  |
| MEAN | 2.66 | 155.00 | 10.48 | 94.40 | 0.98 | 0.60 | 150.20 |
| S.D. | 0.29 | 55.84 | 0.31 | 17.16 | 0.18 | 0.55 | 15.30 |
| N | 5 | 5 | 5 | 5 | 5 | 5 |  |
| GROUP | 25 nm: 100 (㎎/㎏) | | |  |  |  |  |
| MEAN | 2.90 | 113.00 | 10.72 | 96.00 | 1.00 | 0.60 | 142.40 |
| S.D. | 0.22 | 14.70 | 0.28 | 20.68 | 0.10 | 0.55 | 31.62 |
| N | 5 | 5 | 5 | 5 | 5 | 5 | 5 |
| GROUP | 25 nm: 500 (㎎/㎏) | | |  |  |  |  |
| MEAN | 2.83 | 149.00 | 10.53 | 96.25 | 0.95 | 0.75 | 131.25 |
| S.D. | 0.05 | 65.91 | 0.33 | 14.91 | 0.17 | 0.50 | 32.56 |
| N | 4 | 4 | 4 | 4 | 4 | 4 | 4 |

Continued

|  | | |  |  |  |  | |
| --- | --- | --- | --- | --- | --- | --- | --- |
| TESTS: | AST^8^ | ALT^9^ | LDH^10^ | MG^11^ | TP^12^ | UA^13^ | BUN^14^ |
| UNITS: | IU/L | IU/L | IU/L | mg/dL | G/dl | mg/dL | mg/dL |
| GROUP | Control: 0 (㎎/㎏) | | |  |  |  |  |
| MEAN | 79.30 | 38.70 | 106.80 | 2.29 | 6.47 | 1.76 | 14.01 |
| S.D. | 9.83 | 9.60 | 83.82 | 0.20 | 0.36 | 0.60 | 2.09 |
| N | 10 | 10 | 10 | 10 | 10 | 10 | 10 |
| GROUP | 10 nm: 100 (㎎/㎏) | | |  |  |  |  |
| MEAN | 79.60 | 42.20 | 97.80 | 2.32 | 6.50 | 1.28 | 13.96 |
| S.D. | 4.51 | 7.76 | 47.75 | 0.24 | 0.35 | 0.30 | 2.93 |
| N | 5 | 5 | 5 | 5 | 5 | 5 | 5 |
| GROUP | 10 nm: 500 (㎎/㎏) | | |  |  |  |  |
| MEAN | 84.40 | 37.00 | 91.20 | 2.30 | 6.26 | 1.72 | 13.72 |
| S.D. | 13.39 | 7.04 | 53.11 | 0.21 | 0.44 | 0.44 | 1.17 |
| N | 5 | 5 | 5 | 5 | 5 | 5 | 5 |
| GROUP | 25 nm: 100 (㎎/㎏) | | |  |  |  |  |
| MEAN | 207.20 | 58.20 | 410.60 | 2.34 | 6.56 | 1.96 | 13.26 |
| S.D. | 215.68 | 37.05 | 580.63 | 0.15 | 0.40 | 0.67 | 1.69 |
| N | 5 | 5 | 5 | 5 | 5 | 5 | 5 |
| GROUP | 25 nm: 500 (㎎/㎏) | | |  |  |  |  |
| MEAN | 78.75 | 40.50 | 56.00 | 2.10 | 6.73 | 1.08 | 14.85 |
| S.D. | 24.72 | 16.90 | 25.34 | 0.18 | 0.21 | 0.56 | 2.41 |
| N | 4 | 4 | 4 | 4 | 4 | 4 | 4 |

Continued

|  | | |  |  |  |  | |
| --- | --- | --- | --- | --- | --- | --- | --- |
| TESTS: | T-BIL^15^ | IP^16^ | TG^17^ | CPK^18^ |  |  |  |
| UNITS: | mg/dL | mg/dL | mg/dL | U/L |  |  |  |
| GROUP | Control: 0 (㎎/㎏) | | |  |  |  |  |
| MEAN | 0.11 | 9.77 | 58.90 | 102.70 |  |  |  |
| S.D. | 0.04 | 0.16 | 36.19 | 37.53 |  |  |  |
| N | 10 | 10 | 10 | 10 |  |  |  |
| GROUP | 10 nm: 100 (㎎/㎏) | | |  |  |  |  |
| MEAN | 0.13 | 9.68 | 62.20 | 95.40 |  |  |  |
| S.D. | 0.06 | 0.16 | 23.30 | 21.23 |  |  |  |
| N | 5 | 5 | 5 | 5 |  |  |  |
| GROUP | 10 nm: 500 (㎎/㎏) | | |  |  |  |  |
| MEAN | 0.13 | 9.76 | 46.00 | 91.00 |  |  |  |
| S.D. | 0.03 | 0.15 | 16.03 | 20.12 |  |  |  |
| N | 5 | 5 | 5 | 5 |  |  |  |
| GROUP | 25 nm: 100 (㎎/㎏) | | |  |  |  |  |
| MEAN | 0.12 | 9.96 | 41.00 | 906.60 |  |  |  |
| S.D. | 0.03 | 0.18 | 11.07 | 1789.74 |  |  |  |
| N | 5 | 5 | 5 | 5 |  |  |  |
| GROUP | 25 nm: 500 (㎎/㎏) | | |  |  |  |  |
| MEAN | 0.12 | 9.68 | 45.75 | 85.00 |  |  |  |
| S.D. | 0.02 | 0.22 | 19.38 | 22.11 |  |  |  |
| N | 4 | 4 | 4 | 4 |  |  |  |

1, Albumin; 2, Alkaline phosphatase; 3, Calcium; 4, Total cholesterol; 5, Creatinine; 6, Gamma Glutamyl Transpeptidase; 7, Glucose;

8, Aspartate aminotransferase; 9, Alanine aminotransferase; 10, Lactate dehydrogenase; 11, Magnesium; 12, Total protein; 13, Uric acid; 14, Blood urea nitrogen; 15, Total bilirubin 16, Inorganic phosphorus; 17, Triglyceride; 18, Creatine phosphokinase; S.D.: Standard Deviation

N: Number of animals

|  |  |
| --- | --- |

Supplement 23. Number of animal used for biochemical and hematological assays

A. Male

| Gender | Particle size | Dose (mg/kg) | 28 day administration | 1 month recovery | 2 month recovery | Total No. animal |
| --- | --- | --- | --- | --- | --- | --- |
| Male | Control | 0 | 10 | 10 | 10 | 30 |
|  | 10 nm | 100 | 5 | 5 | 5 | 15 |
|  |  | 500 | 5 | 5 | 5 | 15 |
|  | 25 nm | 100 | 5 | 5 | 5 | 15 |
|  |  | 500 | 5 | 5 | 5 | 15 |

B. Female

| Gender | Particle size | Dose (mg/kg) | 28 day administration | 1 month recovery | 2 month recovery | Total No. animal |
| --- | --- | --- | --- | --- | --- | --- |
| Female | Control | 0 | 10 | 10 | 10 | 30 |
|  | 10 nm | 100 | 5 | 5 | 5 | 15 |
|  |  | 500 | 5 | 5 | 5 | 15 |
|  | 25 nm | 100 | 5 | 5 | 5 | 15 |
|  |  | 500 | 5 | 5 | 5 | 15 |

Supplement 24. Number of animal used for tissue content of silver analysis

A. Male

| Gender | Particle size | Dose (mg/kg) | 28 day administration | 1 month recovery | 2 month recovery | 4 month recovery | Total No. animal |
| --- | --- | --- | --- | --- | --- | --- | --- |
| Male | Control* | 0 | 5 | 5 | 5 | 5 | 20 |
|  | 10nm | 100 | 5 | 5 | 5 | 5 | 20 |
|  | 10 nm | 500 | 5 | 5 | 5 | 5 | 20 |
|  | Control* | 0 | 5 | 5 | 5 | 5 | 20 |
|  | 25 nm | 100 | 5 | 5 | 5 | 5 | 20 |
|  | 25 nm | 500 | 5 | 5 | 5 | 5 | 20 |

B. Female

| Gender | Particle size | Dose (mg/kg) | 28 day administration | 1 month recovery | 2 month recovery | 4 month recovery | Total No. animal |
| --- | --- | --- | --- | --- | --- | --- | --- |
| Female | Control* | 0 | 5 | 5 | 5 | 5 | 20 |
|  | 10nm | 100 | 5 | 5 | 5 | 5 | 20 |
|  | 10 nm | 500 | 5 | 5 | 5 | 5 | 20 |
|  | Control* | 0 | 5 | 5 | 5 | 5 | 20 |
|  | 25 nm | 100 | 5 | 5 | 5 | 5 | 20 |
|  | 25 nm | 500 | 5 | 5 | 5 | 5 | 20 |

*, control animals for 28 day administration. 1 and 2 month recovery were used for biochemical and hematological assays, not for 4 month recovery.
